# Mast cells as a unique hematopoietic lineage and cell system: From Paul Ehrlich's visions to precision medicine concepts

**DOI:** 10.7150/thno.46719

**Published:** 2020-08-29

**Authors:** Peter Valent, Cem Akin, Karin Hartmann, Gunnar Nilsson, Andreas Reiter, Olivier Hermine, Karl Sotlar, Wolfgang R. Sperr, Luis Escribano, Tracy I. George, Hanneke C. Kluin-Nelemans, Celalettin Ustun, Massimo Triggiani, Knut Brockow, Jason Gotlib, Alberto Orfao, Petri T. Kovanen, Emir Hadzijusufovic, Irina Sadovnik, Hans-Peter Horny, Michel Arock, Lawrence B. Schwartz, K. Frank Austen, Dean D. Metcalfe, Stephen J. Galli

**Affiliations:** 1 Department of Medicine I, Division of Hematology & Hemostaseology and Ludwig Boltzmann Institute for Hematology and Oncology, Medical University of Vienna, Austria.; 2 Division of Allergy and Clinical Immunology, University of Michigan, Ann Arbor, MI, USA.; 3 Division of Allergy, Department of Dermatology, University of Basel, Basel, Switzerland.; 4 Department of Medicine Solna & Mastocytosis Centre, Karolinska Institutet and Karolinska University Hospital, Stockholm, Sweden.; 5 Department of Hematology and Oncology, University Hospital Mannheim, Heidelberg University, Mannheim, Germany.; 6 Imagine Institute Université Paris Descartes, Sorbonne, Paris Cité, Centre national de référence des mastocytoses, Paris, France.; 7 Institute of Pathology, Paracelsus Medical University Salzburg, Austria.; 8 Servicio Central de Citometria (NUCLEUS), Centro de Investigacion del Cancer (IBMCC; CSIC/USAL and CIBERONC) and Department of Medicine, University of Salamanca, Spain.; 9 Department of Pathology, University of Utah, Salt Lake City, UT, USA.; 10 Department of Hematology, University Medical Center Groningen, University of Groningen, The Netherlands.; 11 Division of Hematology-Oncology and Transplantation, Department of Medicine, University of Minnesota, Minneapolis, MN, USA.; 12 Division of Allergy and Clinical Immunology, University of Salerno, Italy.; 13 Department of Dermatology and Allergy Biederstein, Technical University of Munich, Germany.; 14 Stanford Cancer Center, Stanford University School of Medicine, Stanford, CA, USA.; 15 Wihuri Research Institute, Helsinki, Finland.; 16 Department of Companion Animals and Horses, Small Animal Clinic, Internal Medicine, University of Veterinary Medicine Vienna, Austria.; 17 Institute of Pathology, Ludwig Maximilian University, Munich, Germany.; 18 INSERM UMRS1138, Centre de Recherche des Cordeliers, Paris, France.; 19 Department of Internal Medicine, Division of Rheumatology, Allergy & Immunology, Virginia Commonwealth University, Richmond, VA, USA.; 20 Division of Allergy and Immunology, Department of Medicine, Brigham and Women's Hospital, Harvard Medical School, Boston.; 21 Laboratory of Allergic Diseases, NIAID, NIH, Bethesda, MD, USA.; 22 Departments of Pathology and of Microbiology and Immunology, and the Sean N. Parker Center for Allergy and Asthma Research, Stanford University School of Medicine, Stanford, USA.

**Keywords:** histamine, IgE receptor, *KIT*, mast cell activation, mastocytosis, tryptase

## Abstract

The origin and functions of mast cells (MCs) have been debated since their description by Paul Ehrlich in 1879. MCs have long been considered 'reactive bystanders' and 'amplifiers' in inflammatory processes, allergic reactions, and host responses to infectious diseases. However, knowledge about the origin, phenotypes and functions of MCs has increased substantially over the past 50 years. MCs are now known to be derived from multipotent hematopoietic progenitors, which, through a process of differentiation and maturation, form a unique hematopoietic lineage residing in multiple organs. In particular, MCs are distinguishable from basophils and other hematopoietic cells by their unique phenotype, origin(s), and spectrum of functions, both in innate and adaptive immune responses and in other settings. The concept of a unique MC lineage is further supported by the development of a distinct group of neoplasms, collectively referred to as mastocytosis, in which MC precursors expand as clonal cells. The clinical consequences of the expansion and/or activation of MCs are best established in mastocytosis and in allergic inflammation. However, MCs have also been implicated as important participants in a number of additional pathologic conditions and physiological processes. In this article, we review concepts regarding MC development, factors controlling MC expansion and activation, and some of the fundamental roles MCs may play in both health and disease. We also discuss new concepts for suppressing MC expansion and/or activation using molecularly-targeted drugs.

## Introduction

Mast cells (MCs) are tissue-resident, multifunctional effector cells of the innate immune system that can also substantially contribute to adaptive immune responses. Historically, MCs were described and originally named by Paul Ehrlich in 1879 based on their unique dye-staining properties 1. Ehrlich also described the blood basophil and certain morphologic similarities between both cell types. However, the origin of MCs, and their roles in health and disease, remained mysteries for many decades. Two key clues in defining the functions of MCs were the demonstration that MCs contain histamine and can bind immunoglobulin E (IgE) 2-6. Based on these observations and subsequent preclinical and clinical studies, MCs are now recognized as key effector cells in IgE-dependent allergic inflammation 3-7.

Today, the contributions of MCs to allergic and other inflammatory reactions are well established 3-11. MCs express high-affinity receptors for IgE and produce numerous biologically active substances, some of which are stored in cytoplasmic granules for rapid release 2-4. It is now known that MCs may be activated by many different agents. These include specific antigens (allergens) through allergen-specific IgE and IgE receptors, by certain cytokines, anaphylatoxins, certain neuropeptides, and exogenous toxins, and also by IgG immune complexes and complement, certain drugs, and products of bacteria or other pathogens 2-9, 11. MCs activated by such mechanisms can release their granule-stored mediators, including histamine. In addition, activated MCs may synthesize and secrete many other types of mediators, such as lipid mediators, cytokines and chemokines, and thereby trigger local and systemic reactions 2-7. Although MC activation is observed in a variety of inflammatory, infectious, and other reactive conditions, it has been best characterized during IgE-dependent allergic reactions.

Human MCs have the ability to re-granulate after degranulation and thereby participate in multiple cycles of activation with mediator secretion 8. Moreover, certain MCs resident constitutively within tissues have a remarkably long life span, ranging from months to years, contrasting with the much shorter life span of basophils and other type of granulocytes 9-11. Some MC populations, such as those induced in the gut of mice during infections with certain parasites, can rapidly expand and then drop back to approximately baseline levels upon resolution of the infection 9, 12, 13. Thus, in addition to the long-term, relatively stable MC populations found in many tissues, shorter-lived populations of MCs also appear to exist. These MCs can expand and contract in particular sites in response to local processes, including parasite infections.

However, the rapid expansion of MC populations in multiple organs, including the skin, respiratory system and gastrointestinal tract, is also observed in mice, rats and cynomolgus monkeys injected with the major mast cell growth factor stem cell factor (SCF), also known as KIT ligand 9, 12-14. Upon cessation of SCF injections, the numbers of these expanded MC populations can rapidly return toward baseline values 12-14. These findings, along with the observations that mucosal MCs are absent in T cell-deficient mice, indicate that tissue MC populations may be responsive to changes in ambient levels of soluble SCF, or, perhaps, to changes in membrane-bound SCF or other cytokines.

In this article, we will discuss the progress that has been made over the last decades in addressing the origin and functions of MCs in health and disease, and regarding their neoplastic expansion. We will also discuss current diagnostic and therapeutic approaches to diseases involving MCs, particularly neoplastic disorders.

## Origin of MCs

After their description and naming by Paul Ehrlich in 1879 1, MCs were soon identified as tissue-resident cells in multiple organs, prompting discussions about their origin and functions. It was proposed initially that MCs were derived from blood basophils or from a local histiocytic progenitor. This debate lasted many decades, as no experimental model was available to resolve the issue. However, approximately 100 years after their discovery, the origin of MCs from transplantable hematopoietic stem cells was convincingly demonstrated. This was done in a series of elegant transfer-experiments (and other studies) in mice by Yukihiko Kitamura and his colleagues 15-18. Later, the origin of MCs from transplantable hematopoietic stem cells was confirmed in humans 10. In both species, MCs were found to develop from immature (CD34^+^) hematopoietic precursor cells when these were cultured in the presence of certain cytokines or on stromal cells 19-23.

Subsequent attempts evaluated whether MCs were directly derived from hematopoietic stem cells, from a later progenitor, or even from a mature leukocyte. However, all attempts to grow human MCs from highly purified blood monocytes or blood basophils failed, regardless of the cytokines or culture conditions employed 24. Moreover, in colony assay experiments, human MCs were found to grow preferentially in pure MC colonies or in colonies containing MCs and basophil-like cells 24, 25. Based on these and more recent observations a bi-committed MC/basophil progenitor cell has been postulated, but the existence of such a cell is still under debate. By contrast, some MCs were also detected in mixed colonies derived from pluripotent stem cells 24, 25. Based on these observations, human MCs were considered to derive directly from pluripotent CD34^+^/KIT^+^ stem cells in various organ systems. Such multi-lineage and MC-committed CD34^+^ stem cells are detectable in the bone marrow (BM), peripheral blood (PB) and in the peripheral tissues in most extramedullary organs 9, 22-27.

**Figure 1:**
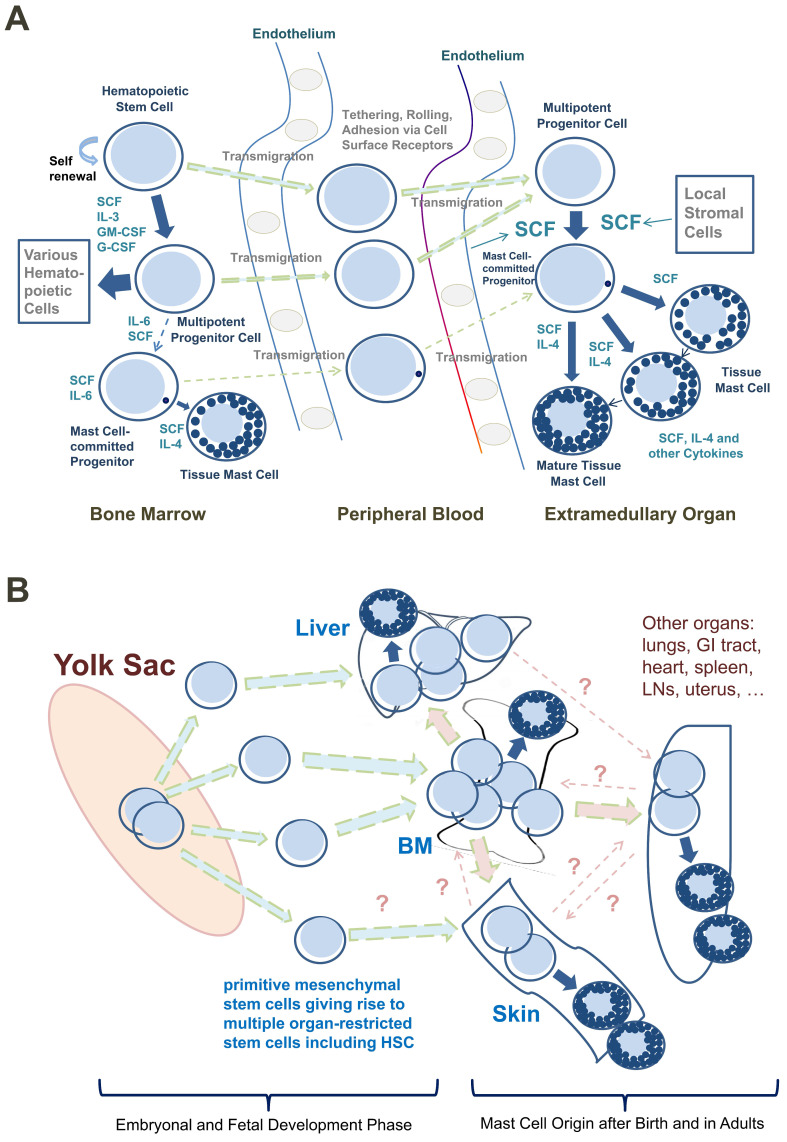
** Development of human mast cells from stem and progenitor cells. A.** Development of mast cells from uncommitted bone marrow-derived stem and progenitor cells. In adult humans, most hematopoietic stem cells (HSC) and mast cell-committed progenitor cells (cells depicted as containing only one cytoplasmic granule in this figure) are considered to originate from the bone marrow (BM) under physiologic (normal) conditions. After leaving the BM, these stem/progenitor cells are detectable in the peripheral blood and after homing to various tissues and exposure to stem cell factor (SCF) these cells undergo maturation and develop into mature mast cells. Note that in the BM, only a few HSC will become mast cell-committed. This is because, apart from SCF, other hematopoietic cytokines are also present and induce differentiation into other (non-mast cell) cell types. **B.** Stem and progenitor cell evolution and distribution during prenatal development. During embryonal and fetal development, primitive (immature) mesenchymal stem cells develop in the yolk sac and give rise to multipotent HSC. Both types of cells may be distributed through the blood stream and can invade other organ systems, including the liver, BM and skin (green arrows). Some of these cells may form a local pool of self-renewing HSC in extramedullary organs before birth and may be able to maintain local pools of HSC and mast cell progenitors via self-renewal in adulthood. After birth, the largest pool of self-renewing HSC is located in the BM. These HSC can leave the BM and can home to extramedullary organs (red colored arrows) where they can undergo mast cell differentiation (blue arrows). However, pools of self-renewing HSC and HSC-derived mast cell progenitors may also originate from yolk sac-derived stem cells and may later persist in adults. These cells develop into mast cells in local organs independent of the BM. There may be additional trafficking routes followed by certain subsets of HSC and mast cell progenitors homing from one to another organ system (dotted line-arrows); however, robust data supporting such HSC trafficking routes in humans are not available. Abbreviations: GI tract, gastrointestinal tract; LNs, lymph nodes.

Based on such observations, most CD34^+^/KIT^+^ MC progenitors are now considered to originate in the BM, to translocate from the BM into the PB, and to enter peripheral tissue sites after homing to various organs (**Figure 1A**) 9, 26, 27. In addition, progenitors with the potential to generate MCs have been identified in the yolk sac 28, 29 and white adipose tissue of mice 30. These latter observations have supported the assumption that BM-independent pools of self-renewing stem cells giving rise to MC progenitors may also develop in local tissue sites early during prenatal development; these cells then can be maintained (via the self-renewal capacity of local stem cells) throughout the lifetime of the organism (**Figure 1B**).

The phenotypic properties of MC-committed progenitor cells have been described in mice and humans 22, 26, 27, 31-37. In humans, these cells express CD34, KIT (CD117), the interleukin-3 receptor (IL-3R) and CD13, but display only low, if any, amounts of FcεRI 31-37. Later, during MC development, MC precursors stop expressing CD34 and the IL-3R alpha chain (CD123) and start expressing higher levels of FcεRI and KIT 27, 34-36. Importantly, MC progenitors display a number of adhesion molecules through which these cells may enter peripheral (extramedullary) organs and tissues. For example, in the mouse, beta7 integrin mediates the adhesion needed for transendothelial movement of BM-derived MC progenitors into peripheral tissues 38.

Despite the many new insights into the origin and development of MCs, several questions remain. First, it is not clear whether human MCs also can be derived from pre-formed local pools of CD34^+^ stem cells within extramedullary organs. Such a local pool of CD34^+^ MC precursors might be established early in life or even during embryogenesis (**Figure 1B**). Another related question is whether MCs in local tissue sites can sometimes emerge from a bi-committed progenitor cell, giving rise to MCs and macrophages or MCs and other leukocytes. In other words, although the direct origin of MCs from blood-born mature monocytes has been formally excluded 24, it remains unknown whether some MCs originate from local bi-committed progenitor cells giving rise to MCs and macrophages at local tissue sites. In this regard, it is worth noting that MCs and macrophages share functional and phenotypic similarities and that advanced systemic mastocytosis (SM) is often accompanied by chronic myelomonocytic leukemia (CMML) 39-42. On the other hand, no association between SM and histiocytic tumors has yet been described. Finally, while definitive information about the exact relationship between human mast cells and basophils remains to be ascertained, most available evidence is consistent with the conclusion that the mature basophil and mast cell lineages are distinct 24, 43, 44.

## Regulation of MC Differentiation, Maturation and Survival

In the mouse, a number of interleukins (ILs), including IL-3, IL-4, IL-9 and IL-10, promote the development of MCs from their BM progenitor cells (**Table 1**) 9, 22, 45-48. However, apart from the support provided by the IL-network, development of mouse MCs is also regulated by tissue-resident stromal cells 9, 20, 21. In a series of pivotal studies, SCF was identified as a major player in stem cell- and MC development and survival in the mouse 9, 12, 26, 49, 50. Deprivation of MC-cultures of SCF (or of SCF-expressing stromal cells) results in MC growth arrest and apoptosis 9, 26. Correspondingly, *Sl/Sl^d^* mice (now designated C57BL/6-*Kitl*^Sl/Sl-d^ mice) lacking (functional) SCF and *W/W^-v^* mice (now designated WBB6F_1_-*Kit*^W/W-v^ mice) lacking a functional SCF receptor, KIT, exhibit a MC-deficient phenotype 9, 15-18, 26.

In 1990, human SCF was cloned and found to act as a ligand of human KIT 51. Shortly thereafter, recombinant human SCF was found to induce the development of MCs from their (CD34^+^/KIT^+^) progenitor cells *in vitro* 52-54. In stromal cells (e.g. fibroblasts and endothelial cells) SCF is often expressed in soluble and membrane-bound forms and can serve as a multifunctional MC regulator. In fact, membrane-bound SCF acts as an adhesion/homing receptor for KIT^+^ stem cells and MCs 9, 11, 26. By contrast, soluble SCF, once released from stromal cells, acts as a MC chemoattractant and as an activation-inducing cytokine 55-62.

In line with these observations, administration of recombinant human SCF to humans resulted in the development of increased numbers of MCs at SCF injection sites 63, 64. In addition, there was evidence for local activation of MCs, specifically reflected in wheal and flare reactions at the skin injections sites in these studies. Moreover, in two participants, systemic signs and symptoms suggestive of anaphylaxis occurred 63, 64. All in all, SCF appears to be a master-regulator of human MCs, promoting the transmigration of circulating MC progenitors into tissues, their local development into mature MCs, the migration and survival of such mature MCs, and MC activation.

Other cytokines may also trigger MC development at certain stages of stem cell differentiation 9, 34, 45-48. In a very early phase of human MC development, multi-lineage cytokines such as IL-3 may expand the pool of CD34^+^/KIT^+^ stem- and progenitor cells and thus facilitate human MC development (**Table 1**) 34-36, 52, 65. A similar effect was also described for IL-6 66-68. Therefore, when applied in early phases of culture, both cytokines can promote SCF-induced MC development *in vitro* 34, 36, 52, 65-68. Similar observations have been made in the mouse system. However, continuous application of IL-3 promotes the differentiation of basophils and other myeloid cells, while downregulating expression of KIT and counteracting differentiation of MCs 69, 70. Correspondingly, late-stage MC progenitors and normal mature, resting human tissue MCs lack IL-3 receptors 34, 36, 71. In these later phases of MC development, other cytokines, such as IL-4, may be involved in the differentiation and maturation of MCs 72-74. In particular, IL-4 has been described as triggering expression of chymase, certain adhesion molecules, and high-affinity IgE receptors in human MC precursor cells 72-75. Depending on the environment and organ, IL-4 may even promote the proliferation of maturing human MCs 75. However, IL-4 has also been described as downregulating KIT expression and KIT-dependent differentiation of human MC precursor cells and causing apoptosis of human MC progenitors and of lung MCs that express only tryptase (MC_T_), unless IL-6 is also present 70, 76-78.

As mentioned, many different ILs and other cytokines can be involved in MC differentiation, maturation, and survival in the mouse. These cytokines can trigger the expression of multiple proteases in MCs and their progenitors, thus influencing the cells' phenotype, and/or can influence expression of molecules that enhance MC survival 12, 79-82. It seems likely that all of these cytokines act in concert to promote MC differentiation and maturation in various tissues and organs 9, 11, 80, 81, 83. Since cytokine-expression depends on the tissue-type and pathology of the affected organ, certain MC phenotypes appear to be tissue-dependent and pre-determined, for example by the cytokine-network characteristics of the underlying condition and/or pathology 7, 9, 11, 80, 81, 83. In addition, MC phenotypes may change depending on the organ system and the local tissue microenvironment 9, 11, 83-87. **Table 1** provides an overview of the effects of cytokines on the development and maturation of mouse and human MCs.

The signal transduction pathways and effector molecules downstream of MC cytokine receptors have been analyzed extensively and a detailed description is beyond the scope of this review. However, a few important signaling nodes and effector molecules should be mentioned, as these are now also recognized as potential targets of therapy. One important target appears to be the PI3-kinase/AKT/mTOR pathway 88-91. Disruption of this pathway is associated with a decreased ability of MC precursors to develop into mature MCs and also impairs MC activation 88-91. The RAS/MEK/ERK pathway is also potentially important for MC development and function 92-94. Finally, the JAK-STAT5 pathway has been implicated in KIT-dependent growth and survival of MCs 91, 95. All three pathways may be highly active, particularly in neoplastic MCs exhibiting the transforming KIT mutant D816V. Depending on additional mutations (some of them directly affecting these pathways, like oncogenic *RAS* mutations), one, two, or all three of these pathways seem to play important roles in oncogenesis and drug resistance 91, 95.

Among several survival molecules acting downstream of AKT, mTOR, RAS or STAT5, members of the BCL2 family (such as MCL-1, BAX or BCL-xL, and several heat shock proteins, like heme oxygenase-1, HSP70 and HSP90) play critical roles in the survival of normal and/or neoplastic human MCs 96, 97. Effects of such anti-apoptotic molecules may not only explain SCF-dependent survival of normal MCs, but also the accumulation of neoplastic MCs in patients with mastocytosis 96. Indeed, MCs in such patients express excess amounts of these survival-promoting molecules, and pharmacologic inhibition of these survival molecules is associated with reduced survival and increased apoptosis in neoplastic MCs 96. **Table 2** provides a list of critical signaling and survival molecules relevant to KIT-dependent growth and survival in normal and neoplastic MCs.

Finally, a number of transcription factors are considered to be involved in the development and differentiation of mouse MCs, including, among others, GATA2, microphthalmia (mi) transcription factor (MITF), and STAT5 98-102. A role for STAT5 in the growth of neoplastic human MCs has also been postulated 91, 95. Whether the transcription factors GATA2 and MITF also play a role in the development or function of normal and/or neoplastic human MCs remains unknown.

## MC Deficiency Models: Historical Aspects and Recent Developments

As outlined above, the development and differentiation of human or mouse MCs from their uncommitted hematopoietic stem- and progenitor cells depends largely on the presence of a functional KIT receptor and its ligand, SCF. KIT is a tyrosine kinase receptor that acts as a facultative onco-protein and regulates the growth, differentiation and survival of germ cells, hematopoietic stem cells, MCs, interstitial (intestinal pacemaker) cells of Cajal (ICCs), and melanocytes. In mice, ´loss-of-function´ of *KIT* (for example in WBB6F_1_-*Kit*^W/W-v^ mice) results in macrocytic anemia, a profound MC deficiency, deficiency of germ cells, lack of ICCs, depigmentation of the skin, and failure of neutrophils to express inhibitory leukocyte immunoglobulin (Ig)-like receptor B4 9, 15, 26, 103-105. Apart from *Kit-*deficient mice, there are a number of additional MC deficiency models that implicate specific target pathways and molecules 106. MC deficiency also may be due to a lack of functionally active SCF (also known as steel factor, SL), for example in *Sl/Sl*^d^ (C57BL/6-*Kitl*^Sl/Sl-d^) mice 16, 106. These mice express only the extracellular, non-membrane associated, domain of SCF. Another model of MC deficiency is based on the lack of a functional MITF 106, 107. MITF is involved in the regulation of expression of KIT in hematopoietic stem cells, MC progenitors and melanoblasts, and is itself regulated by KIT activity 106, 107. In addition, MITF regulates the production of a number of mediators, including specific proteases in MCs and MC progenitor cells 108-113.

Initial studies investigating MC functions *in vivo* principally focused on MC-deficient mice whose MC deficiency was due to diminished function of KIT, such as in *Kit*^W/W-v^ mice, or decreased expression of KIT, such as in *Kit*^W-sh/W-sh^ mice. The MC deficiency of *Kit*^W-sh/W-sh^ mice is based on a large genetic inversion upstream of the c*-kit* locus which disrupts corin 114 and results in a profound MC deficiency as well as other phenotypic abnormalities, including increased levels of neutrophils and basophils 115, 116. Both *Kit*^W/W-v^ mice and *Kit*^W-sh/W-sh^ mice develop tissue MC populations after adoptive transfer of MCs derived *in vitro* from the hematopoietic cells of the corresponding wild type (WT) mice or from other normal or genetically-altered mice of suitable strains, thus producing 'mast cell knock-in mice' 117. After adoptive transfer, such *in vitro*-derived MC populations, which often consist of BM -derived cultured MCs (BMCMCs), gradually acquire multiple phenotypic characteristics which resemble those attributed to the native MC populations in the corresponding anatomical sites of WT mice 84-86, 117. Accordingly, *Kit*^W/W-v^ mice and *Kit*^W-sh/W-sh^ mice engrafted with *Kit* WT MCs or MCs bearing specific genetic abnormalities have been used to investigate the functions of MCs and certain MC receptors or products in diverse biological responses and models of disease 115.

However, as detailed elsewhere 117-119, such experiments are subject to several admonitions, including: 1) the phenotypes and anatomical distribution of the adoptively-transferred MCs at diverse anatomical sites may not be identical to that of the corresponding native MC populations in WT mice, particularly after intravenous injection of such *in vitro*-derived MCs 117; 2) the reality that a biological response in *Kit*^W/W-v^ or *Kit*^W-sh/W-sh^ mice can be “normalized” after the adoptive transfer of MCs to such mice indicates that MCs can express the detected function in the context of those mutant mice (which have multiple phenotypic abnormalities in addition to their profound MC deficiency), but does not by itself prove that MCs are necessary for the same functions in WT mice; and 3) the limited strain background of the *Kit*^W/W-v^ or *Kit*^W-sh/W-sh^ mice used for early studies of MC function may have revealed some MC roles that are strongly expressed in mice on the C57BL/6 or related backgrounds, but may be expressed weakly, if at all, on other strain backgrounds, such as BALB/c 119.

Given such concerns about the general utility of *Kit*^W/W-v^ or *Kit*^W-sh/W-sh^ mice for studies of MC function, several groups have attempted to generate transgenic mice that express MC deficiency by mechanisms independent of those reflecting abnormalities in KIT or its ligand. As reviewed elsewhere 115, a variety of mouse strains have now been developed which constitutively or inducibly lack, or are deficient in, some or all MC populations by KIT-independent mechanisms. While each of these more recent models of MC deficiency has abnormalities in addition to the MC deficiency (e.g., two of these strains have moderately reduced numbers of basophils 120, 121), these non-MC abnormalities are less pronounced than those in *Kit*^W/W-v^ and *Kit*^W-sh/W-sh^ mice. The availability of these newer strains of MC-deficient mice should further advance our understanding of the roles of MCs in mammalian biology.

To date, there has been no description of humans with a genetically-determined MC deficiency. However, humans with a single loss-of-function mutation in *KIT* can exhibit the clinical picture of piebaldism, a rare autosomal dominant disorder characterized by symmetrical pigment defect reflecting a localized lack of melanocytes in the skin and lack of melanin in the hair shafts. This condition may present as a patch of white hair (poliosis) in the forehead and/or a patch of non-pigmented amelanotic skin (leukoderma) 122, a pigment phenotype much like that of mice with a single loss-of-function mutation in c-*Kit* 9, 15, 106. Although the piebald phenotype also can reflect loss-of-function mutations in genes other than *KIT* (e.g., *SNAI2* 123), piebaldism was arguably the first genetic transmitted pathology recognized in recorded history by the ancient Greeks. In these patients, the ability of hematopoietic progenitor cells to differentiate into MCs in response to SCF may be slightly impaired (P.V., personal observation). However, based on the ´mosaic-pattern´ of the defect, no complete MC deficiency is found in these patients. Although some patients with AIDS or various genetically-determined immunodeficiencies may exhibit reduced numbers of MCs in the gastrointestinal mucosa 124, no other natural or clinical model of MC-deficiency has yet been identified in humans.

However, it has been described that patients with chronic myeloid leukemia (CML) receiving imatinib, a strong KIT inhibitor, for 2 or more years can develop a profound MC deficiency 125. The long latency until MC deficiency occurs in these patients is best explained by the long time required by MC progenitors to develop into mature MCs and the long life-span (years) of some populations of mature MCs 9, 10, 26, 34, 36. Given the evidence that the development of human tissue MCs from their progenitor cells is critically dependent on SCF and KIT, it is not unexpected that long-term treatment with strong inhibitors of KIT-activation can produce MC deficiency. The clinical relevance of such an induced MC-deficiency, however, remains to be determined.

## Proteomic and Genomic Expression Profiling Confirms that MCs form a Unique Lineage within the Hematopoietic Cell System

It is generally acknowledged that MCs are distinct hematopoietic cells that display a unique profile of leukocyte differentiation antigens 40, 43. Since tissue MCs display a number of myeloid determinants, these cells are commonly considered to be ´myeloid cells´ 40, 43. Initially, protein profiling of human MCs was largely restricted to biochemical investigations and antibody-typing. Thus, between 1989 and 2005, extensive antibody-typing was performed to establish the phenotype of primary human tissue MCs obtained from various organs 40, 43, 126, 127. In all organs tested, MCs were found to display substantial amounts of CD9 and KIT (CD117) as well as certain myeloid surface antigens, including CD13 and CD33. The phenotype of primary tissue MCs was also compared to that of blood basophils and blood monocytes. These studies revealed that many other myeloid cell surface structures, like CD11b, CD14, CD15, or CD35, otherwise displayed by basophils and/or monocytes, are not expressed, or expressed only at trace amounts, on human MCs 40, 43, 128.

**Figure 2:**
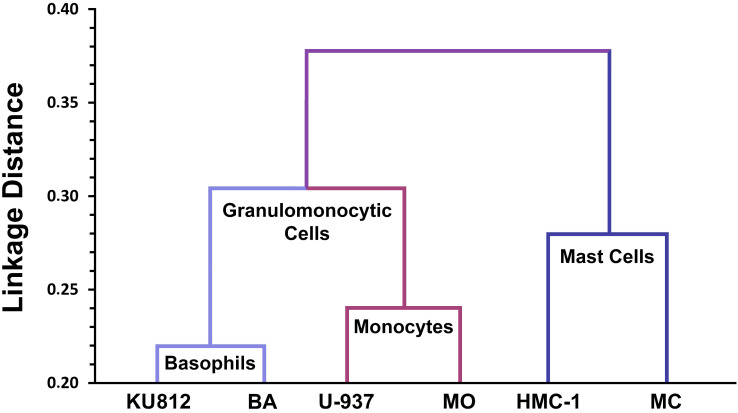
** Lineage relationships between human mast cells, basophils and monocytes based on CD antigen expression profiles.** Human lung mast cells (MC), the human mast cell line HMC-1, normal blood basophils (BA), the human basophil cell line KU812, normal blood monocytes (MO) and the human monoblastic cell line U-937 were analyzed using a panel of 90 different CD antibodies provided by the Leukocyte Typing Workshops. Based on antibody-reactivities, linkage distance analyses were performed (Agis H, et al., Immunology. 1996; 87: 535-43). As expected, the linkage distance within the cell lineages examined (primary cells versus respective cell lines) is low. Primary basophils and monocytes and the respective lines were also found to be related phenotypically. By contrast, however, the phenotype of MC did not reveal a close relationship with BA or MO, neither in the cell line context nor in primary cells (primary MC vs primary BA). These data suggest that MC and BA form two separate (independent) hematopoietic cell lineages within the leukocyte family.

All in all, a detailed phenotypic comparison between MCs, monocytes and basophils, and their respective cell lines was consistent with the conclusion that MCs are neither related to basophils nor to blood monocytes (**Figure 2**) 128. Later large-scale genomic studies, transcriptome analyses, and proteomic studies confirmed that the mRNA and protein expression profiles of MCs are unique and distinct within the family of hematopoietic cells 129-131. Finally, *KIT* mutational studies in patients with systemic mastocytosis (SM) confirmed that MCs form a distinct cell lineage without a direct developmental relationship with basophils or other leukocytes 44.

The notion that MCs form a separate hematopoietic cell lineage defined by specific cell surface antigens has major practical and clinical implications. First, the unique phenotype of MCs enables their detection and their enrichment from various tissues 43, 126, 127, 132. Second, the counting and phenotyping of MCs critically contribute to the diagnostic work-up of patients with suspected SM 133-135. In this context, it is noteworthy that the phenotype of neoplastic MCs in patients with SM is aberrant, recurrent, and unique. In particular, in contrast to MCs in normal or reactive tissues, MCs in SM usually display CD25 and often also CD2 and/or CD30 133-136. Currently, two of these antigens, CD2 and CD25, serve as minor diagnostic criteria for SM defined by the World Health Organization (WHO) 137-139. The fact that these aberrantly expressed antigens are otherwise primarily expressed on lymphopoietic cells, has recently re-introduced a discussion about the nature and origin of normal and neoplastic MCs and, more importantly, re-enforced the concept that MCs form a unique hematopoietic cell lineage.

There are also additional antigens that are expressed aberrantly on neoplastic MCs, such as the IL-3 receptor alpha chain (CD123) 140. Other cell surface antigens, including CD203c and the C5a receptor (C5aR) CD88, are usually expressed at higher levels on neoplastic MCs in SM compared to normal BM MCs 134, 141, 142. Interestingly, some of these receptors are also expressed on 'reactive (activated) MCs' in various inflammatory conditions. For example, in patients with rheumatoid arthritis, MCs can express the C5aR CD88 143. Even CD25 may be detectable in reactive MCs in inflamed tissue sites (P.V., unpublished observation). Therefore, these surface antigens serve as minor, not major, criteria of SM. **Table 3** provides a summary of relevant cell surface molecules expressed on normal, reactive and neoplastic MCs 43, 135, 144.

## Mediators and Other Compounds Produced by Tissue MCs

MCs contain many functionally defined and clinically relevant mediators in their cytoplasmic (secretory) granules 3, 4, 7, 145-152. In human MCs, these include, among others, histamine, heparin, tryptase, carboxypeptidase, and chymase, which are stored in their metachromatic granules (**Table 4**). Other mediators, such as lipids (e.g. prostaglandin D2, leukotriene C4, platelet-activating factor, and sphingosine-1-phosphate), are primarily produced during MC activation. In general, MC-derived mediators are classified on the basis of their chemical structures and their specific biological functions, as well as their locations of storage or origin within MCs. Granule-derived mediators are often bound to heparin and many of them contribute to inflammatory (allergic) reactions 151-153. Some MC-derived mediators, such as heparin, tissue type plasminogen activator (tPA), MC proteases and MC-derived cytokines (some of which appear to be pre-formed), are molecules characteristically associated with local tissue repair 154-159.

*In vitro*-derived mouse MCs also can produce a wide variety of cytokines, chemokines and growth factors 11, 146-150 and some of these products also have been detected in isolated mouse peritoneal mast cells 11. While some of these products apparently can be stored in certain MC granules, such as tumor necrosis factor (TNF) alpha or vascular permeability factor (VPF), also known as vascular endothelial growth factor (VEGF), the largest amounts of these substances are generally thought to be synthesized and directly secreted after MCs have been functionally activated 11, 146-150, 159, 160.

By contrast only a few cytokines and chemokines have been detectable in resting human MCs. In particular, normal human tissue MCs are considered to store some preformed TNF-alpha in their granules. However, normal human MCs do not express many other cytokines unless activated by external stimuli. By contrast, after IgE-dependent activation *in vitro* or exposure to activating cytokines such as SCF, human (and mouse) MCs can express and release several different cytokines, including interleukins (like IL-3) and chemokines 146-150, 160. Most of these cytokines and chemokines are also produced in neoplastic MCs, where the activating KIT mutant D816V acts as a persistent stimulus for cytokine/chemokine synthesis. For example, *KIT* D816V-transformed neoplastic MCs display substantial amounts of oncostatin-M (OSM), IL-8 and the CC-chemokine ligand 2 (CCL2) also known as monocyte chemotactic protein 1 (MCP1), and CCL23 161, 162. However, other studies suggest that normal human MCs, either in the resting state or after activation via the FcεRI, do not generate multiple cytokines 129, 160, 163. These observations highlight the need for additional research to ascertain the ability of normal vs. neoplastic human MCs to generate cytokines and chemokines under different conditions and states of activation.

**Table 4** presents a summary of clinically relevant MC-derived mediators and cytokines. A comprehensive overview of all these molecules is beyond the scope of this article. However, it should be emphasized that several MC mediators, such as histamine, are clinically important as they contribute to the typical signs and symptoms of patients with IgE-dependent or independent allergies and patients with mastocytosis. Other mediators may be involved in tissue remodeling and repair processes, including fibrosis and angiogenesis 7, 152, 153, 161, 162. Whether some of the specific signs and symptoms observed in advanced SM, such as weight loss (an established TNF effect) or fatigue, are triggered by MC-derived cytokines (e.g., during cytokine storm) is presently unknown.

## Mechanisms Contributing to MC Mediator Release and Releasability

The ability of MCs and basophils to respond to IgE-dependent or -independent stimuli (i.e. the cells´ “releasability”) is determined by many factors. These include genetic and epigenetic effects, the type and dose of agonist (e.g., specific allergen recognized by the cells' FcεRI-bound IgE antibodies), the local micro-environment, the levels of ambient cytokines (which may either directly induce mediators secretion or modify the cell's responses to other stimuli), and the underlying disease pathology 164, 165. The features and severity of reactions evoked by MC activation depend on additional factors, such as the type and amounts of mediators released and the number and location of reacting MCs (and other immune cells including basophils) involved in the response 166, 167. When MC activation is substantial, this can result in systemic anaphylaxis and, in certain settings, the criteria for a MC activation syndrome (MCAS) may be fulfilled 168, 169. However, MCs also can undergo activation chronically or locally in a number of inflammatory conditions without clinical signs or symptoms of systemic MC activation, so that MCAS criteria are not fulfilled 170.

In IgE-dependent allergic disorders, IgE-receptor cross-linking is usually the critical event that leads to mediator release 4-7. Certain cytokines promote such IgE-dependent reactions. In humans, SCF augments MC IgE-dependent histamine release 60-62. When applied at higher concentrations and/or for longer periods of time (>90 minutes at ≥100 ng/ml), SCF can directly induce histamine secretion in primary human tissue MCs 60-62. Similar effects of SCF have been described in mice *in vivo* 57 and in humans injected subcutaneously with recombinant human SCF 63. In those settings, SCF induced degranulation that resembled IgE-induced anaphylactic degranulation 57, 63. However, it is worth noting that the activation of MCs by certain cytokines can differ substantially from anaphylactic degranulation. In particular, ultrastructural analyses have shown that the stimulation of MCs with certain cytokines results in piecemeal degranulation in which vesicles are thought to transport mediators to the cell surface in the absence of classical degranulation and in which the slow release of mediator substances follows. In contrast, IgE-dependent activation is usually followed by rapid degranulation and the release of stored MC mediators via the process of rapid compound exocytosis 171.

Apart from SCF and IgE-dependent stimuli, many other agonists also induce mediator release in human MCs. For example, C3a and C5a induce histamine release in human CD88^+^ skin MCs 172-174, and in a small subset of CD88^+^ lung MCs that express both tryptase and chymase (MC_TC_ type) 175. In many other extra-cutaneous organ systems, MCs usually lack CD88 and do not respond to C3a or C5a unless an inflammatory reaction is ongoing, such as in rheumatoid arthritis 143. In the mouse, genetic and MC transfer studies indicate that C3a and C5a, acting via the C3aR and the C5aR respectively, can directly induce degranulation of skin MCs and that the endogenous production of such anaphylatoxins may also augment the intensity of IgE-dependent MC activation in the skin 176.

Several seminal review articles have discussed the various biochemical and signal transduction pathways that underlie IgE-receptor-mediated, cytokine-induced or C3a/C5a-mediated activation, degranulation, and mediator release in MCs 2-6, 9. **Figure S1** provides an overview of IgE-receptor-dependent and KIT-dependent signaling cascades. For more details, the reader is referred to the available literature. It is important to note that several of these activation-linked events and downstream signaling molecules are emerging novel targets of therapy for allergic (atopic) diseases, mastocytosis and MCAS.

## Heterogeneity and Versatility of MCs

Several observations suggest that mouse and human MCs in various organs have heterogeneous phenotypic and functional properties 9, 79-81, 83, 87, 135, 143, 172-175, 177-179. Despite earlier contributions by William Bate Hardy 179 and others, Lennart Enerbäck is often regarded as the first to provide a detailed description of MC heterogeneity in rodents 177. He identified two distinct types of MCs in rat tissue sections: mucosal MCs (MMCs) and connective tissue-type MCs (CTMCs) 177. Compared to CTMCs, MMCs are smaller, more variable in shape, and often hypo-granulated. CTMCs were also identified as a unique source of heparin. By contrast, rodent MMCs contain glycosaminoglycans of lower sulfation, no heparin, low amounts of histamine, and little or no serotonin 177. A surprising observation was that special fixation procedures were necessary to detect MMCs in rat tissue sections. Later, MMCs and CTMCs were also detected in mice.

In both species, MMCs and CTMCs display specific patterns of MC proteases 180-182. Notably, mouse MMCs preferentially contain mouse MC protease (mMCP)-1 and -2, whereas CTMCs express mMCP-4, -5, -6, and carboxypeptidase A 180-182. In addition, it was found that the development of MMCs and CTMCs is controlled by distinct cytokines, including SCF (CTMCs), IL-3 (MMCs), and other MC-targeting interleukins 12, 46, 79-81, 83, 182. CTMCs were also regarded as innate or constitutive MCs whereas MMCs were thought to represent an adaptive MC compartment, which is inducible by the tissue microenvironment in response to inflammatory processes 183.

Subsequent studies revealed that the phenotypes of MMCs and CTMCs are, at least in part, reversible in certain tissue locations and cytokine milieus, and that trans-differentiation between the two types of MCs may occur in physiologic and pathologic tissues 81, 83-87, 182. In addition, based on the expression of various combinations of different MMCPs, MC heterogeneity is complex and cannot be fully understood based on a simple model predicting two major MC subsets (MMCs and CTMCs). Rather, increasing data suggest that MCs are functionally and phenotypically versatile cells. In fact, MCs may change their phenotype (e.g. protease-composition) rapidly depending on the cytokines to which these cells are exposed, the tissue microenvironment, the maturity of the responding MC population, and the underlying pathology 81, 83-87, 143, 182.

In humans, MC heterogeneity was first described on the basis of differential expression of neutral proteases in MCs derived from the skin and other organs. In particular, it has been reported that skin MCs express both tryptase and chymase in their secretory granules (MC_TC_), whereas most MCs in the lung and small intestinal mucosa display tryptase but not chymase (MC_T_) 178. Attempts to relate MC_T_s to mouse MMCs and MC_TC_s to CTMCs were only partly successful, mostly because mixtures of MC_T_s and MC_TC_s were found in most extra-cutaneous tissue sites.

Human MCs can also be classified based on expression of certain surface receptors and responses to certain stimuli 143, 172-175. For example, the C5aR CD88 is expressed abundantly on all MCs derived from skin (MC_TC_s) and on the small portion of lung MCs that are MC_TC_s, but not detectably on human lung MC_T_s 174, 175. Expression of the C5aR on MCs may also depend on the underlying disease. For example, the C5aR CD88 is expressed on synovial MCs in rheumatoid arthritis but not on synovial MCs in patients with osteoarthritis 143. Carboxypeptidase A3, which is usually expressed only in MC_TC_s in healthy tissues, is also expressed in chymase-deficient MCs in certain inflammatory sites, including eosinophilic esophagitis, severe asthma, exercise-induced bronchospasm and chronic sinusitis. Finally, opioid receptors, including the MRGPRX2 receptor, are expressed on human skin MCs, but not on MCs in other organs 163, 184.

To date, the factors and mechanisms underlying expression of CD88 and carboxy-peptidase A3 in MCs remain unknown. One hypothesis is that chronic cytokine exposure or continuous KIT activation triggered by a mutation leads to expression of CD88. In line with this hypothesis, MCs in patients with *KIT* D816V^+^ SM constitutively display CD88 185. Other markers are also expressed on both neoplastic MCs in SM and reactive MCs in inflamed tissues. One interesting surface marker-antigen is the alpha-chain of the IL-2 receptor (IL-2RA), CD25. This antigen is usually not expressed on MCs in healthy tissues. However, in pathologic tissues and chronic inflammatory reactions, MCs may display CD25 (P.V. unpublished observation). Again, the factors responsible for abnormal expression of CD25 on MCs remain unknown.

Another important observation is that MC antigens may be displayed differentially based on the cells´ stage of maturation. For example, the high affinity IgE receptor, FcεRI, is not expressed on very early MC precursors but appears later during MC maturation 34-36, 53, 67, 68. Finally, certain cell surface antigens are rapidly expressed, or increase, upon IgE-dependent or cytokine-induced activation of MCs. Similarly, compared to resting MCs, IgE receptor-activated MCs express increased amounts of CD63 and CD203c 135, 142. It has also been described that interferon-gamma induces the expression of HLA-DR and CD64 (FcγRI) on human MCs 186, 187. Finally, in patients with mastocytosis, neoplastic MCs express various cell surface antigens, including CD25, CD30, CD32, CD64, CD88, CD123, CD203c, and HLA-DR in an augmented or aberrant manner 133-136, 138-142.

## Potential Physiologic and Pathologic Role(s) of MCs

During the past few decades, several attempts have been made to decipher the possible physiologic roles of MCs, one of the last unresolved riddles about these cells. While no definitive solution to this quandary has been presented, a number of interesting possibilities have been suggested. One evolving hypothesis is that MCs are physiologically involved in orchestrating tissue repair processes, in a variety of settings, through which the evolution of certain pathologies can be avoided. This role would therefore be similar to that of neutrophils and macrophages in combatting bacterial and other infections. In fact, during the past 30 years, more and more data support the probability that MCs play an important role as defense and repair cells during various pathologic conditions and related biological processes. Another emerging concept is that MCs and their products are essentially involved in the innate, and acquired, defense against the lethality of animal-derived venoms, a concept that has recently been established experimentally in mice and may also apply to humans. These concepts, and the diverse roles MCs may play in the destructive and healing processes of various tissues, are discussed in the following paragraphs.

## Potential Role(s) of MCs as Defenders against Microbes and Toxins

Several findings point to the possible roles of MCs as defenders in certain infectious diseases 188-194. While a complete overview of all proposed functions of mast cells in infectious diseases is beyond the scope of the current article, we here discuss a few important concepts that relate to the role of MCs as possible contributors to host defense. One remarkable observation was that MC-deficient mice were more susceptible to certain fatal bacterial infections, such as peritoneal infection and septicemia following cecum ligation and puncture 189, 190. Similar observations have been made in other models of bacterial infections 116, 188. It had also been described that the protective 'MC-effect' depends on the rapid availability of MC-derived TNF-alpha, a cytokine known to augment the influx of phagocytes into inflamed tissue sites 189, 190. Indeed, exposure of endothelial cells to MC-derived histamine and TNF-alpha promotes leukocyte rolling, leukocyte adhesion, and the consecutive transmigration of phagocytes. In the absence of such phagocyte-trafficking, any bacterial disease would become potentially life-threatening.

However, other studies performed in MC-deficient *Kit*^W/W-v^ and *Kit*^W-sh/W-sh^ mice suggested that MC-derived TNF-alpha may actually contribute to an adverse outcome in certain severe forms of cecal ligation and puncture 116. More recent data suggest that, in the mouse, basophils represent a significant contributor to survival in a moderate form of cecal ligation and puncture, and that this role may at least in part depend on basophil production of TNF-alpha 195. Other studies have shown that bacterial antigens directly induce the release of mediators, including TNF-alpha, from MCs 191-193. However, it remains unknown how much TNF-alpha can be provided by human MCs in reactions such as these. A number of additional mechanisms may contribute to the ability of MCs in various organs to enhance resistance to bacterial infections 188, 194, 196, 197. These mechanisms may include, among others, MC activation via Toll-like receptors (TLR)s 194 (although TLRs have not been identified on human skin MCs by all groups 163), complement activation 196, and activation through endothelin-1 198. Moreover, both human and mouse MCs have been implicated in bacterial phagocytosis 194, 197.

On the other hand, several observations argue against a major role for human MCs as defenders against bacterial infections. First, human MCs are not capable of compensating a severe neutropenia to rescue septicemic patients, e.g. during high-dose chemotherapy (where MC usually survive). Second, in patients with imatinib-induced MC deficiency, no increase in the rate of total or severe bacterial infections has been reported 125. These observations do not totally exclude the possibility that MCs contribute to the immunological defense system against bacteria or other microbes in humans. However, compared to other immune cells, in humans the contribution of MCs appears to be less important.

MCs have also been implicated in host defense against various parasites. For example, in mice, an intact MC system seems to be required for proper defense against certain helminth infections, such as infections by *Strongyloides ratti*, *Strongyloides venezuelensis* or *Trichinella spiralis* 199-201. In addition, mouse MCs may contribute to host defense against primary cutaneous infections with *Leishmania major* 202.

A similar role of MCs as defenders against parasites in humans has not been established to date. In this regard, it is worth noting that the physiologic and pathogenetic role of MCs in host defense against pathogens may have changed during mammalian evolution. In mice and rats, MCs may be part of a strong defense-system against bacteria, helminths, and other pathogens. However, in humans, it is possible that these MC functions may no longer be as critically required to guarantee host survival. On the other hand, evidence favoring a protective role for MCs in response to *Strongyloides stercoralis* has been described in non-human primates 203.

Finally, MCs have been implicated in the etiology of several viral infections. In fact, a number of viruses have been reported to be capable of infecting human MCs, including the human immunodeficiency virus (HIV) 204-206. MCs are unique among HIV-vulnerable cell types in that they appear to only be susceptible to infection with R5-tropic HIV for a brief period during their ontogeny when they are CD4^+^/CCR5^+^/CXCR4^+^ 204. It also has been reported that IgE-FcεRI interactions enhance the susceptibility of these MC precursors to HIV 205. As these infected MCs enter into tissues, mature, and persist, they appear to provide a long-lived, inducible, reservoir of persistent HIV in tissues. *In vivo* evidence supports this possibility, in that HIV-infected women have both circulating precursor MCs and placental tissue MCs that harbor inducible infectious HIV even after having been treated during their pregnancy with highly active antiretroviral therapy (HAART) 206. Other viruses are also reported to infect human MCs. For example, the LAD2 MC line and primary human cord blood-derived MCs have been infected with human rhinoviruses and RSV 207, 208. In addition, the corona virus receptor CD26 is expressed on human skin MCs 131.

MCs also may be able to contribute to immune defense mechanisms against viruses. For instance, rodent, monkey, and human MCs are able to detect dengue virus (DENV), a single-stranded, positive-sense RNA virus, which results in MC activation and degranulation 209. This MC response has been linked to the MC-driven recruitment of natural killer and natural killer T cells. Similarly, human MCs can produce type I IFNs after exposure to double-stranded RNA and/or respiratory syncytial virus or reovirus type 1, the former via specific interactions with TLR-3 208. Such observations are consistent with the conclusion that MCs can contribute to innate immune responses to viral infections in part via the production of type I IFNs.

Recently, MCs and their products also have been implicated in the defense of mice against animal-derived venoms 210-216. In fact, several findings in MC-deficient, MC-reconstituted, or MC chymase-deficient mice indicate that mouse MCs (and for certain venoms, mouse MC chymase), can enhance the survival of mice challenged for the first time with the venom of the honeybee, several snakes, a lizard, or two scorpions 210, 212, 215. As a consequence, the venom-induced tissue damage and the resulting death of the host, are significantly ameliorated 210, 212, 215. In the case of honeybee and Russell's viper venoms, IgE-dependent MC activation can further enhance resistance to the lethality of these venoms in mice 213, 215. Moreover, zebra fish embryos were used to assess the toxicity of snake venoms treated with human MC proteases 217. It was determined that human MC tryptase-ß, but not human MC chymase or CPA, could reduce the toxicity of the venoms of six poisonous snakes 217. Taken together, this evidence indicates that MCs, MC proteases, and IgE may play important protective roles against the serious and indeed fatal, effects of animal-derived venoms. However, so far, no broadly applicable therapeutic approach has been developed using MC proteases.

## Potential Role of MCs as Repair Cells in IgE-Dependent Allergic Reactions

A number of observations point to important 'repair-roles' of MCs during allergen-induced anaphylaxis 210, 218-220. First, MC-derived proteases have been described as degrading several different allergens into inactive fragments 218, 219. Moreover, MC-derived proteases have been reported to degrade IgE 220. The functional consequence of this MC-derived protease activity would be a disruption of the allergen-induced tissue damage. All of these data point to important biological feedback mechanisms during human allergic reactions in which MCs and MC-derived products can help to limit the local and systemic reactions to allergens. In line with this concept, MC-derived proteases can also degrade a number of cytokines, chemokines, and vasoactive peptides into inactive fragments.

## Potential Role of MCs as Repair Cells in Thromboembolic Disorders and Wound Healing

Several biochemical and functional studies in humans and other species suggest that MCs represent the major source of heparin 9, 154, 158, 221. It has also been reported that MC-deficient *Kit*^W/Wv^ mice develop more severe thromboembolic events after India ink injection compared to their normal littermates, and that injection of heparin can rescue these mice from fatal thromboembolic events 222. In addition, human MCs are a unique source of non-complexed tissue-type plasminogen activator (tPA) 157. Whereas in many other cell types, tPA is expressed and released together with its natural inhibitors (PA inhibitors = PAIs) and in the absence of heparin, MCs produce and release un-complexed (active) tPA together with its stabilizer and activator, heparin 157. In addition, it has been found that MCs accumulate in areas of thromboembolism, especially around thrombosed vessels 223, and that thrombin-activated endothelial cells express and release soluble SCF, which in turn attracts MCs (**Figure S2**) 224.

These observations support the hypothesis that MCs can act as major repair cells in thromboembolic disorders, and that in physiologic tissues MCs may even be integrated in a prophylactic repair system that counteracts thromboembolic events 157. Other studies have shown that patients with clonal MC disorders may suffer from a bleeding tendency, and that in these patients, hyper-heparinemia and sometimes even hyperfibrinolysis (caused by elevated tPA) may be detected 225-228.

However, several questions remain. For example, it remains unknown which triggers cause liberation of heparin and tPA from MCs in patients with MC disorders. It also remains unknown whether MC-derived heparin and tPA can indeed prevent local thromboembolic events. Finally, no increase in thromboembolic events has been observed in CML patients receiving life-long imatinib, although these patients develop a MC deficiency 125. This observation suggests that other cells and mechanisms can counteract thrombosis. One possibility is that basophils may counteract some of the effects of a MC deficiency.

MCs have also been implicated as repair cells in wound healing 155, 156, 229-232. This hypothesis is based primarily on the observation that MCs can produce several important repair molecules, including heparin, proteases, and angiogenic cytokines, such as VEGF, fibroblast growth factor (FGF) and IL-6 155, 156, 159, 229-231. In addition, MCs represent a potentially rich source of oncostatin-M (OSM), IL-8 and CCL2 161, 162. In several experimental models, there is evidence that MCs indeed may contribute to wound healing 229-231. However, an unequivocal role of MCs in wound healing could not be confirmed in all of the model systems examined 232, 233.

## Possible Role of MCs in Atherosclerosis and related Cardiovascular Diseases

Several observations in experimental animals and in man suggest that MCs may play an important role in the evolution and progression of atherosclerosis 234-249. Early studies revealed that activated MCs can support the generation of cholesterol-filled macrophages (foam cells) which is typical finding in atherosclerosis 234. Mechanistically, the apolipoprotein B-100 (apoB-100) component of low-density lipoprotein (LDL) particles binds to the heparin proteoglycan component of exocytosed MC granules. Subsequently, MC granule-derived chymase can degrade apoB-100, thereby inducing fusion and tight binding of the LDL particles to the granules 236. Ultimately, the LDL-loaded granules are phagocytosed by macrophages, thereby leading to cholesterol accumulation in these cells 234, 235. MC granules can also facilitate uptake of LDL by smooth muscle cells and thereby promote their transformation into foam cells 238. Chymase and tryptase were also later found to degrade the apolipoprotein A-I component of high-density lipoprotein (HDL) 243. This renders the HDL particles unable to remove cholesterol from macrophages, thereby accelerating cholesterol accumulation and foam cell-formation in the arterial wall 243.

By immunohistochemistry, MCs containing tryptase and chymase have been detected in the normal intimal layer of healthy human aortas, as well as in the fatty streaks and vulnerable shoulder regions of atheromas in atherosclerotic aortas 237. Moreover, studies in human coronary arteries revealed the accumulation of activated MCs at the actual site of erosion or rupture of the culprit coronary plaque that was responsible for an acute coronary syndrome or myocardial infarction 240, 241.

Mechanistic insights into the possible roles of MCs in plaque erosion and rupture have also been obtained from *in vitro* culture experiments. In these studies, human MC-derived proteases were found to degrade the pericellular matrix of endothelial cells and, together with TNF-alpha, induce their apoptotic death 245, 247. This process is known to lead to de-endothelialization of the plaque and thus to plaque erosion. Moreover, MC-derived proteases can degrade the pericellular matrix of smooth muscle cells, with apoptotic cell death resulting from loss of matrix-derived (external) survival signals 242. Loss of these collagen-producing cells may in turn result in further thinning of the collagenous cap of the plaque, thereby increasing the risk of plaque rupture 244. Thus, MC-derived proteases may significantly contribute to the erosion and rupture of an atherosclerotic plaque, both by directly degrading pericellular matrices and by facilitating degradation of the extracellular matrix via activation of matrix metalloproteinases 246.

Finally, MCs and their products have been implicated in inflammatory processes that trigger and sustain atherogenesis 249. All of these observations point to potentially important roles of arterial MCs and their inflammatory products in cholesterol accumulation in the vessel wall and in plaque vulnerability and thus in the progression of atherosclerosis. In addition, MC-engraftment of the meninges of genetically MC-deficient *Kit^W/Wv^* mice has provided evidence that meningeal MCs can worsen stroke pathology in that species, perhaps in part through the release of IL-6 250. Supplemental **Figure S3** shows some potential mechanisms by which activated MCs may contribute to various stages of atherogenesis.

However, several questions remain concerning the role of MCs in atherosclerosis. First, systemic disorders characterized by a marked chronic activation of MCs, or increases in MC numbers, such as atopic disorders, severe allergies or mastocytosis, are not known to be associated with an increased risk of atherosclerosis, myocardial infarct or stroke. In addition, multi-kinase inhibitors targeting KIT lead to MC deficiency 125 but cannot protect the individual from the development of atherosclerosis. Indeed, some of these kinase blockers (nilotinib and ponatinib) promote atherosclerosis and the occurrence of thromboembolic events 251. On the other hand, the mechanisms through which these TKI promote atherosclerosis are not well understood and may involve several different cell types and molecules. Moreover, imatinib induces MC deficiency but does not promote the development of atherosclerosis in patients with CML 125.

MCs have also been proposed as providing some protective functions in the context of atherosclerosis. First, MCs provide heparin together with un-complexed (PAI-free) tPA 157, a 'master-cocktail´ that is used clinically to treat patients with acute arterial occlusion such as myocardial infarction or ischemic stroke. Moreover, heparin released from activated MCs in atherosclerotic lesions may restrict the development of a platelet-rich arterial thrombus on the de-endothelialized (eroded) surface of an atherosclerotic plaque. MCs also provide several additional repair molecules, such as FGF, VEGF, OSM, or IL-8 155-159, 161, 162. These MC-derived repair molecules may contribute to neo-angiogenesis and re-perfusion of ischemic organs (after infarction) as well as re-canalization of thrombosed vessels 157.

From these observations, the hypothesis has been raised that MCs may exert both pro-atherogenic and anti-atherogenic activities, as well as both pro- and anti-repair effects, during ongoing atherosclerosis. In reality, the net effect of MCs and MC-specific effector-molecules may largely depend on additional factors, including the involved vessel site and the stage of atherosclerosis. For example, the fibrinolytic and fibrinogenolytic activities of MC proteases (tPA, tryptase) may counteract atherosclerosis and related vascular events in small and large arteries, at both early and later stages of atherosclerosis. On the other hand, the effects of these molecules may also be fatal, for example when the thinned fibrous cap of an atheroma ruptures. **Table 5** summarizes the potential harmful and protective effects of various MC products in atherosclerosis.

## Role of MCs as Inflammatory Effector Cells in Allergic and Immunologic Diseases

Previous reports have described the pivotal roles of MCs in IgE-dependent and IgE-independent inflammatory reactions 2-6. Although a detailed review cannot be provided in this article, we should note that the key event in such reactions is the release of pre-formed granule-derived and newly generated pro-inflammatory and vasoactive mediators, cytokines, and chemokines 2-7, 145-150. These mediators and cytokines are major triggers of immediate and late-phase reactions and thus of the clinical symptoms in allergic disorders. Depending on many factors, most of which regulate the releasability of MCs (and basophils), the reactions and symptoms range from mild to severe or even life-threatening 164-169.

However, as mentioned, MCs can also be involved in regulatory feed-back mechanisms that can help to limit allergic reactions and symptoms 210-212, 218-220. Desensitization against IgE-dependent and IgE-independent stimuli also has been described 252-254. Many of these mechanisms may also apply in patients with systemic mastocytosis (SM), where the excessive number of MCs results in an increased risk for life-threatening anaphylaxis in a subset of patients. However, many patients with SM maintain a clinically stable course without any mediator-related symptoms, so that the hypothesis has been raised that their neoplastic MCs may be less responsive to IgE-dependent and other stimuli through desensitization phenomena and other negative-feedback mechanisms 255.

On the other hand, many patients with SM suffer from recurrent systemic anaphylaxis or even MCAS 167-169. In these patients, as well as in those with severe allergies (without SM), the regulatory mechanisms described above may have been bypassed or are too weak to keep the reaginic proinflammatory machinery under control. Recent data suggest that patients with severe allergy symptoms, or those with mastocytosis and severe symptoms, may have extra copies of their alpha tryptase gene, a genetic condition called hereditary alpha tryptasemia (HAT) 256, 257. However, not all of these HAT patients have severe symptoms and in many cases, the exact mechanisms that cause severe anaphylaxis in SM and/or MCAS remain unknown. Regarding therapy, patients with severe MCAS are treated symptomatically with drugs targeting MC mediators or their receptors, such as histamine receptor blockers, MC-stabilizing drugs and other anti-allergic medications. In severe forms, antibodies targeting IgE, such as omalizumab, may be considered.

Until approximately 1980, MCs were primarily regarded as key effector cells of allergic reactions. However, since then MCs and their mediators have been implicated in various other inflammatory conditions and immunologic disorders 7, 9, 143, 150, 258-264. For example, MCs have been described as contributing to pro-inflammatory reactions and processes in patients with various systemic auto-immune disorders 58, 143, 258-264. While a complete overview of the MC´s potential involvement in these disorders is beyond the scope of this article, we wish to emphasize one of the most prominent and important examples, the impact of MCs on the pathogenesis and evolution of rheumatoid arthritis (RA).

Earlier studies had shown that MCs are detectable in the inflamed synovial tissue of the affected joints in patients with RA and that cytokines, such as TNF-alpha, which can be derived from MCs and other participants in these disorders, play a pivotal role in the pathogenesis of the disease 258-261. TNF-alpha and other (potentially) MC-derived cytokines have also been implicated in the recruitment and activation of leukocytes into the inflamed joints in RA 258. Subsequently, SCF produced by activated synovial fibroblasts and other local cells was described as attracting and activating MCs, thereby providing a mechanism through which MCs accumulate and release their cytokines in synovial areas in RA 59.

However, other MC agonists, such as C3a and C5a, as well as IgE and other immunoglobulins, may also be involved in MC recruitment and activation in RA 143. Other studies have shown that MC-derived proteases play a crucial role in the pathogenesis of RA 262, 263. For example, among several different effects on local effector cells of RA, MC-derived tryptase can augment the survival and activation of local (synovial) fibroblasts 263. In addition, MC-derived proteases may augment autoimmune responses in RA 262.

The most critical MC-derived cytokine in RA appears to be TFN-alpha. Since this cytokine (which can be derived from MCs and other cell types) can activate synovial cells to produce collagenase, a key enzyme that can induce destruction of cartilage, antagonists of TNF-alpha have indeed been shown to have beneficial effects in this disease 265. Other therapeutic attempts have aimed at the direct suppression of MC growth or MC activation in patients with RA 260. However, to date, no robust therapeutic concept focusing solely on the MC compartment has been described in the context of RA or other autoimmune disorders. Also of note, recent work with both two types of MC-deficient mice and human synovium, has implicated MCs, and IgE/FcεRI and Syk, in the development of osteoarthritis 266.

## Potential Role of MCs in Tumor Development and Tumor Surveillance

A number of different studies have pointed to several potential roles MCs may play in tumor development and/or tumor surveillance 267-271. In fact, depending on the tumor type, tissue of origin, experimental model, and biologic context, MCs have been thought either to promote or inhibit the development of a neoplasm 267-271. The mechanisms underlying this proposed dual role of MCs is not completely understood. However, MCs can produce a number of substances that may contribute to tumor or leukemia cell proliferation and survival (e.g., growth factors), tumor cell redistribution and metastasis (e.g., vasoactive substances, chemotactic factors, adhesion receptor-inducers) and/or modulation of the tumor-related microenvironment (e.g., angiogenesis-promoting compounds) 145-156, 159. Many MC-related mechanisms and molecules may play a role in immune surveillance against tumor growth. These include, among others, TNF, perforin/cytolysin, proteases, and IgE-dependent processes that lead to tumor cell destruction 272, 273. Unfortunately, most such observations have been made in experimental models, and the actual net impact of MCs during tumor development in patients suffering from various malignancies remains unknown. It is also worth noting that chronic MC activation is not known to be associated with a lower or higher risk of human malignancies.

## Neoplastic Disorders of MCs and their Precursors

The pathologic accumulation of MCs in the skin of patients with urticaria pigmentosa (UP) was first described by Paul Gerson Unna in 1887. Later, the term mastocytosis was proposed, based on the stable clinical course of most patients and the belief that the MC accumulations were reactive rather than neoplastic in nature (the suffix 'osis' is usually employed to describe benign/reactive expansions of leukocytes). This hypothesis was supported by the observation that the cutaneous lesions of mastocytosis, when manifested in early childhood, often disappeared during or shortly after puberty 274.

In 1949, Ellis described the first case of systemic mastocytosis (SM) with involvement of internal organs 275. From this and other similar cases it was appreciated that mastocytosis in adults is a persistent and neoplastic disease that may be complicated by organ involvement or even organ damage. At approximately the same time, the first cases of MC leukemia (MCL) were described. Since then, a bone marrow biopsy has been regarded as a basic standard in the diagnostic evaluation of patients with suspected SM.

A first comprehensive classification of mastocytosis was proposed by Karl Lennert and the Kiel group in 1979 276. In 1988, Travis et al. proposed a classification for systemic mast cell disease which introduced indolent, aggressive and associated hematologic disease categories 277. In 1991, Dean Metcalfe proposed a first consensus classification of mastocytosis 278. The clonal nature of SM was confirmed by demonstrating that the involved cells (MCs and their precursors) exhibit the transforming *KIT* mutation D816V 279-281. This mutation had been described as one of two activating *KIT* mutations in the HMC-1 mast cell line that was derived from a patient with MCL 282, 283. Shortly afterwards, *KIT* mutation analysis was introduced as a major diagnostic test in patients with suspected SM.

Between 1990 and 2000, specific morphologic, immunologic, and molecular features of neoplastic MCs were examined for their specificity and robustness. Based on such studies, criteria were proposed to make the diagnosis of SM and to support a related classification of mastocytosis 137. This proposal serves as a basis of the WHO classification of mastocytosis 138, 139. The most recent update of this classification divides the disease into cutaneous mastocytosis (CM), indolent SM (ISM), smoldering SM (SSM), SM with an associated hematologic neoplasm (SM-AHN), aggressive SM (ASM), MCL, and MC sarcoma (MCS) 138, 139. We refer to the available literature for the details of the classification, and for the criteria of SM. However, it is essential to focus here on a few aspects related to the clinical impact of MCs in these disorders.

First, it is important to recognize that patients with SM may or may not present with severe mediator-related symptoms. Patients who are suffering from such symptoms and require continuous treatment with anti-mediator-type drugs are labeled as SM_SY_ 169. In severe cases, a MCAS is almost always detected in these patients 168, 169. Others suffering from SM also have an underlying hypertryptasemia (HAT). Another important point is that in pediatric patients, in whom the disease was long believed to be a ´reactive´ (non-neoplastic) process, the disorder is now regarded as a neoplastic condition, because in most cases activating (somatic or germline) mutations in *KIT* are detectable 284, 285. Remarkably, however, the presence of the D816V *KIT* mutation or other *KIT* mutations cannot predict the clinical course in pediatric CM. Rather, it is the presence of the typical (adult-like, i.e. small sized, monomorphic) form of the cutaneous lesions that helps predict persistence of pediatric mastocytosis into adulthood 286.

Another riddle regarding MCs in SM was the fact that the *KIT* D816V mutation is expressed in MCs in both indolent SM associated with a normal life-expectancy, as well as in MCs in advanced SM, including MCL where the survival time is usually very short 137-139, 279-281, 285. In this regard, it is important to appreciate that *KIT* D816V acts as weak oncogene regarding proliferation but as a strong inducer of MC differentiation, maturation and survival, processes that are usually associated with reduced proliferation 287. However, in a subset of patients with SM, *KIT* D816V can be detected in MCs, as well as in other hematopoietic lineages 288, 289. Such multi-lineage involvement is typically found in smoldering SM and advanced SM and is of prognostic significance 290.

Moreover, several additional oncogenic mutations (apart from *KIT* mutations) have been detected and serve as new diagnostic and prognostic parameters in patients with advanced SM (SM-AHN, ASM or MCL) 291-295. The current hypothesis is that these additional mutations (most commonly in: *TET2*, *SRSF2*, *ASXL1*, *CBL*, *RUNX1*, and *RAS*) act together with *KIT* D816V in advanced SM to trigger proliferation and survival of neoplastic MCs. The type and number of such additional mutations have a major impact on the prognosis (including overall survival and responses to therapy) of patients with SM 295. For example, a particularly poor prognosis is found in patients who carry mutations in* SRSF2*, *ASXL1*, and *RUNX1* (S/A/R-patients) 295. It is also worth mentioning that most of these mutations are also found in other myeloid neoplasms (without SM) and that, among advanced SM patients, they are most frequently detected in SM-AHN.

Taken together, the unique features of SM and the cell-specific evolution of MC progenitors and other cell lineages in advanced SM provide additional evidence that MCs form a separate myeloid lineage within the hematopoietic cell system. Moreover, despite expression of certain ´lymphoid´ antigens on neoplastic MCs, the progression-patterns seen in patients with SM-AHN confirm that MCs are best regarded as myeloid cells. In fact, in almost all patients with SM-AHN, the AHN portion of the disease manifests as a myeloid neoplasm, but only rarely as a lymphoid malignancy 41, 137-139.

## New Treatment Concepts: Targeting of MC Growth and/or Activation

During the past 3 decades, several different treatment approaches have been established for MC proliferative disorders and for conditions defined by MC activation. Whereas initial attempts to block MC growth in patients with advanced SM were based on treatment with conventional anti-neoplastic drugs, like interferon-alpha or cladribine 296, 297, more recent approaches are based on using validated drug targets 138, 298-302. Considering the possibility of inducing MC deficiency by blocking MC development from their progenitors, the clinically most relevant targets identified so far are WT KIT (normal MCs) and the KIT mutant D816V that acts as a driver of MC development and differentiation in CM and SM 255, 283, 284, 287. As mentioned before, imatinib is a strong KIT inhibitor and can induce an almost complete MC deficiency when administered continuously 125. MC-depletion in subjects treated with imatinib takes about 2 years 125. Application of such KIT inhibitors also may be of interest in the context of MC activation, atopic disorders or severe allergies. Indeed, a recent double-blind, placebo-controlled study in a small number of patients with severe asthma indicates that imatinib may be useful in this setting, although it is not clear to what extent the benefit observed was due to effects on MCs as opposed to other actions of the drug 298, 299.

However, the *KIT* D816V mutation confers imatinib resistance on neoplastic MCs 300, 301. Therefore, novel KIT tyrosine kinase inhibitors have been developed for testing in preclinical and clinical studies. One of these drugs is midostaurin (PKC412), which blocks the kinase activity of wild type KIT, KIT D816V and several other clinically relevant KIT mutants 300, 301. In addition, in contrast to most other KIT blockers, midostaurin inhibits SYK activation and several other IgE-receptor downstream targets; midostaurin thereby blocks MC activation and IgE-dependent mediator secretion in MCs and basophils 302.

In a first pilot patient with advanced SM, midostaurin was found to block the expansion of *KIT* D816V-mutated MCs 303. More recently, midostaurin was administered to patients with advanced SM in a global, single arm, phase II trial. In this study, midostaurin was found to induce major and often durable clinical responses in many cases with advanced SM, including MCL 304. Remarkably, many patients not only developed meaningful hematologic responses but also major responses in mediator-related symptoms and a substantial increase in their quality of life 304. The latter effect of the drug may reflect its ability to suppress mediator release in MCs 302. Based on these data, midostaurin was approved by the US Food and Drug Administration and the European Medicines Agency in 2017 for all subtypes of advanced SM 305. In addition, midostaurin may be an interesting drug for patients with non-advanced SM in whom mediator-induced symptoms cannot be controlled by conventional anti-mediator type drugs.

In addition to midostaurin, several other KIT-targeting drugs have been tested in the context of advanced SM 301. Whereas some of them, like imatinib or masitinib, are unable to suppress the kinase activity of mutant KIT (especially D816V), others, like avapritinib or ripretinib, are potent inhibitors of KIT D816V and can block the growth of neoplastic progenitor cells 306. As noted above, all of these inhibitors are candidates for evaluation in the context of allergic/atopic disorders and various immunologic or rheumatologic diseases where MC activation is thought to play an important role 298, 307. However, in these non-neoplastic diseases, the use of these agents must be carefully balanced against their side effects. It should also be kept in mind that some of the clinical effects and side effects of the 'KIT-targeting' drugs may reflect actions on KIT expressed by cells other than MCs or on tyrosine kinases other than KIT. Other drugs act on various signaling or effector molecules downstream of KIT and/or downstream of the IgE receptor 308, 309. The clinical efficacy of many of these agents is currently being explored in clinical trials. Finally, for patients with severe allergies and MCAS, and for those who are suffering from severe allergies, MCAS and/or additional aggravating MC conditions (like HAT and/or mastocytosis), application of antibodies removing IgE, such as omalizumab or ligelizumab, or targeting MC tryptase, may represent promising additional approaches 310-314. Finally, a number of inhibitory receptors, such as Siglec-7, Siglec-8, or CD300a, have been identified on human MCs and are currently being validated for their role as therapeutic target sites 315-318.

## Concluding Remarks and Future Perspectives

Since their description and naming by Paul Ehrlich, MCs have been the subject of intensive research and have fascinated generations of scientists. Some of the riddles concerning the origin and functions of MCs have now been solved. MCs are hematopoietic cells derived from CD34^+^ stem cells, develop and survive under the influence of SCF, and can undergo neoplastic transformation based on acquiring gain-of-function mutations in the *SCF receptor* (*KIT*) gene and other critical target genes. Moreover, some of the mechanisms underlying MC activation in various disease states and related syndromes have been defined. Also, a variety of diagnostic markers and drug targets have been identified and have been successfully validated, and several drugs targeting MC expansion, MC activation, or both have been translated from preclinical testing into clinical applications. In advanced MC disorders, KIT appears to be a most promising target. Finally, diagnostic criteria for MC disorders, including mastocytosis and mast cell activation syndromes, have been established.

However, many questions remain. For example, there is still debate about the possible roles of MCs in healthy tissues. Notably, the long-term MC-deficiency induced by persistent treatment with imatinib in humans has not yet been associated with a particular adverse phenotype. Also, in several pathologic conditions, it remains unknown whether MC activation may be useful, e.g., by enhancing repair processes, is harmful and leads to tissue damage, or, depending on the circumstances, may contribute to either outcome. Finally, it remains unknown which KIT-independent molecular pathways and molecules play the most decisive role in the progression of mast cell neoplasms. We trust that future MC research will provide answers to such questions, leading to a better understanding of MC biology in health and disease, as well as to the development of new and better diagnostics and better treatments for patients suffering from MCAS, advanced MC neoplasms or other MC-dependent pathologies.

## Supplementary Material

Supplementary figures and tables.

## Figures and Tables

**Table 1:** Effects of cytokines on growth and differentiation of mouse and human mast cells

	Effects on proliferation (p) or maturation (m) of:			
	Mouse*		Human**	
Cytokine	MC precursors	MCs	MC precursors	MCs
SCF	+ (p,m)	+ (p,m)	+ (p,m)	+ (m)
IL-3	+ (p,m)	+ (p,m)	+ (p)	-
IL-4	+ (p,m)	+ (p,m)	+/- (p,m)***	+/- (p,m)***
IL-5	+ (p,m)	+ (p,m)	-	-
IL-6	+ (p)	+/- (p,m)	+ (p)	-
IL-9	+ (p,m)	+ (p,m)	-	-
IL-10	+ (p,m)	+ (p,m)	-	-
IL-11	-	-	-	-
IL-12	-	-	-	-
IL-13	+ (p,m)	+ (p,m)	n.k.	+/- (p)
IL-15	+ (p,m)	+ (p,m)	n.k.	n.k.
IL-33	+ (p)	+ (p)	n.k.	+ (m)
GM-CSF	+ (p)	-	-	-
NGF	+ (p,m)	+ (m)	-	-

Abbreviations: MC, mast cell; SCF, stem cell factor; (p), proliferation; (m), maturation; IL, interleukin; n.k., not known; GM-CSF, granulocyte-macrophage colony-stimulating factor; NGF, nerve growth factor. *In most studies mouse mast cell precursors were obtained from the bone marrow and studies on mouse mast cells refer to data obtained with IL-3-dependent bone marrow-derived mast cells. **Most studies on human MC precursors were performed using cord blood cell progenitors, and most studies on mature MCs were performed using isolated human lung mast cells, skin mast cells, or mast cells cultured from their cord blood progenitors using IL-3, SCF and IL-6. ***Under certain conditions, IL-4 can even induce apoptosis in MCs and MC precursor cells.

**Table 2:** Critical signaling and survival molecules relevant to KIT-dependent growth and survival in normal and neoplastic human mast cells

Expressed in resting and/or activated human mast cells (MCs)				
Molecule	Resting MCs	FcεRI cross-linked MCs	SCF-activated MCs	KIT D816V+ neoplastic MCs
**Phosphorylated (p) signaling molecules**				
pERK	-	+	+	++
pAKT	-	+	+	++
pS6	-	+	+	++
pSTAT5	-	+	+	++
pBTK	-	+	+/-	+/-
**Survival-related anti-apoptotic molecules**				
BCL-2	+/-	+/-	+	+
MCL-1	-	-	+/-	++
BCL-xL	-	-	+/-	++
BIM	-	-	-	-
BAX	-	-	-	-
PUMA	-	-	-	-
NOXA	-	-	-	-
HO-1/HSP32	-	-	+/-	++
HSP70	-	-	+	++
HSP90	-	-	+	++

Abbreviations: MCs, mast cells; STAT5, signal transducer and activator of transcription 5; BTK, Bruton´s tyrosine kinase; HO-1, heme oxygenase 1.

**Table 3:** Cell surface antigens expressed on normal, reactive and neoplastic human mast cells

Cell surface antigens expressed on normal, reactive and neoplastic human mast cells						
		Expressed on the surface of:				
Antigen	CD	Lung MCs*	Skin MCs*	HMC-1	MCs in ISM**	MCs in MCL**
LFA-2	CD2	-	-	+/-	+/-	-/+
AMP-N	CD13	+	+	+	+	+
LPS-R-r	CD14	-	-	-	-	-
IL-2RA	CD25	-	-	+/-	+	+
Ki-1	CD30	-	-	+/-	+/-	+/-
Siglec-3	CD33	+	+	+	+	+
HPCA-1	CD34	-	-	-	-	-
Leukosialin	CD43	+	+	+	+	+
Hermes-R	CD44	+	+	+	+	+
CLA	CD45	+	+	+	+	+
ICAM-1	CD54	+	+	+	+	+
LFA-3	CD58	+	+	+	+	+
LAMP-3	CD63	+	+	+	+	+
C5aR	CD88	-/+	+	+	+	+/-
GM-CSFRA	CD116	-	-	-	-	-
KIT	CD117	+	+	+	+	+
IL-3RA	CD123	-	-	-	-	+/-
L1CAM	CD171	n.k.	+	+/-	n.k.	n.k.
ENPP3	CD203c	+/-	+/-	+/-	+	+
FcεRI	n.c.	+	+	-	+	-/+
MRGPRX2	n.c.	-	+	-	n.k.	n.k.

*Lung mast cells (MCs) from surgical tumor tissue samples and skin MCs from inflamed foreskin samples were examined. **Mast cells in patients with ISM and MCL usually express the *KIT* mutation D816V. All data were obtained from the available literature. Abbreviations: CD, cluster of differentiation; ISM, indolent systemic mastocytosis; MCL, mast cell leukemia; LFA-2, leukocyte function-associated antigen-2; AMP-N, aminopeptidase-N; IL-2RA, interleukin-2 receptor alpha chain; HPCA-1, human precursor cell antigen 1; LAMP-3, lysosomal associated membrane protein-3; C5aR, complement component 5a receptor; Ki-1, Kiel-antigen-1; CLA, common leukocyte antigen; ICAM-1, intercellular adhesion molecule-1; GM-CSFRA, granulocyte-macrophage colony-stimulating factor receptor alpha chain; ENPP3, ectonucleotide pyrophosphatase/phosphodiesterase 3; n.c., not (yet) clustered; n.k., not known.

**Table 4:** Selection of biologically relevant mediators that human mast cells produce and secrete

Substance	Biologically relevant functions
Histamine	Vasodilation, vascular permeability, endothelial cell priming for leukocyte-rolling, neuroendocrine mediator, pro-inflammatory
Heparin	Co-factor of ATIII, of tPA and of tryptase, anti-inflammatory
PGD_2_	Induces bronchoconstriction, activates endothelial cells, induces vasodilation and VEGF production, activates Th2 lymphocytes, eosinophils and basophils
Alpha tryptase	May promote mast cell activation
Beta tryptase	Fibrinogenolysis, mitogen for fibroblasts and endothelial cells, lipid-modifier, degrades VIP, endothelin, fibronectin, collagen, calcitonin gene‐related peptide, protease‐activated receptor 2, RANTES and eotaxin
Chymase	Lipid-modifier, degrades apolipoprotein B, VIP, fibronectin, vitronectin, bradykinin, HGF, SCF, C3a, and thrombin; induces smooth muscle cell and endothelial cell apoptosis, converts angiotensin (Ang) 1 to Ang 2, and big endothelin to endothelin, activates IL-1-beta and TGF-beta-1
TNF-alpha	Endothelial cell and macrophage activation, induces apoptosis in smooth muscle cells and other perivascular cells, mediator of catabolic processes, tissue inflammation and tissue damage
IL-3	Multipotent growth factor for myeloid cells, expands the pool of multilineage progenitor cells in the bone marrow, induces the differentiation of basophils, eosinophils, and macrophages, promotes activation of (primes) basophils and eosinophils
IL-8	Induces chemotaxis (migration) and activation of granulocytes and monocytes, pro-inflammatory and angiogenic mediator
CCL2	Induces leukocyte chemotaxis and activation
OSM	Promotes angiogenesis, fibrosis and tissue remodeling
VEGF	Promotes angiogenesis and vascular permeability
FGF	Promotes fibrosis and wound healing

ATIII, anti-thrombin 3, tPA, tissue type plasminogen activator; PGD_2_, prostaglandin D_2_; VEGF, vascular endothelial growth factor; VIP, vasoactive intestinal peptide; RANTES, regulated on activation, normal T cell expressed and secreted; HGF, hepatocyte growth factor; SCF, stem cell factor; IL, interleukin; TGF, transforming growth factor; TNF, tumor necrosis factor; CCL2, CC-chemokine ligand 2; OSM, oncostatin M; VEGF, vascular endothelial growth factor; FGF, fibroblast growth factor.

**Table 5:** Potential contributions of mast cells (MCs) and their products to biological processes relevant to the development of atherosclerosis

Effects	MC mediators involved
**A. Proatherogenic effects**	
Proinflammatory effects in the vessel wall	TNF-alpha and other MC-derived cytokines
Recruitment of leukocytes/phagocytes into the vessel wall	Histamine, MC-derived cytokines (TNF-alpha and others) and chemokines (CCL2, IL-8, others)
Modification and cellular uptake of LDL by macrophages and smooth muscle cells consecutive foam cell formation	Tryptase and chymase as well heparin
Modification of HDL molecules into pathologic species that are no longer capable of mobilizing cellular cholesterol	Tryptase and chymase
Endothelial cell and smooth muscle cell death with subsequent plaque rupture	MC proteases and TNF-alpha
Leakage and disruption of microvessels in neovascularized plaque areas	Histamine, tryptase and chymase
**B. Protective effects and repair functions**	
Thrombolysis: degradation of fibrin and fibrinogen	tPA (fibrin-degrader) and uPAR; Tryptase (fibrinogen-degrader); Heparin (co-factor for tPA, tryptase and anti-thrombin III)
Protection of endothelial cell layers and vessel wall integrity	Tryptase (endothelial cell survival) and angiogenic cytokines (VEGF, CCL-2, IL-8, others)
Neovascularization of hypoxic regions in atherosclerotic plaques	Angiogenic cytokines (VEGF, FGF)

Abbreviations: tPA, tissue type plasminogen activator; VEGF, vascular endothelial cell growth factor; FGF, fibroblast growth factor.
