# Supplementary material for: Mast cells as a unique hematopoietic lineage and cell system: From Paul Ehrlich's visions to precision medicine concepts
Source: Theranostics. 2020 Aug 29;10(23):10743–68. doi: 10.7150/thno.46719 (PMC7482799; doi:10.7150/thno.46719)
Supplement: Supplementary file 1 — Supplementary figures and tables. [file thnov10p10743s1.pdf]

Supplemental (S) Figures – Figure Legends

Figure S1

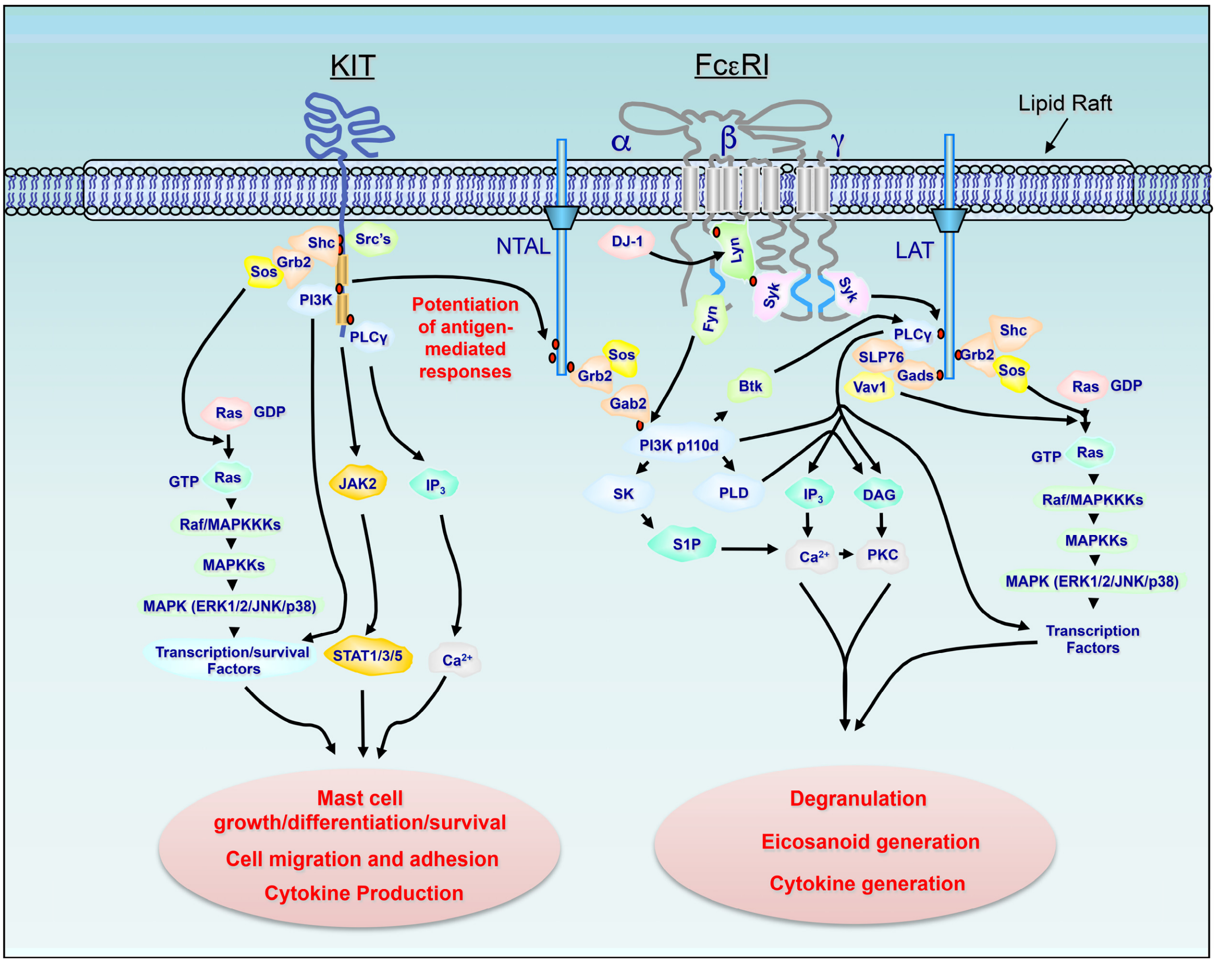

Signal transduction downstream of the stem cell factor (SCF) receptor KIT, and FcεRI, that contribute to mast cell functions in health and disease

Binding of SCF to KIT results in homodimerization of KIT (not shown in this figure), followed by activation of KIT´s intrinsic tyrosine kinase activity and auto-phosphorylation of tyrosine residues in its cytosolic tail. These phosphorylated residues serve as "docking sites" for distinct signaling molecules, including SRC kinases, SHC, phosphoinositide 3-kinase (PI3K), and phospholipase Cγ (PLCγ). Activation of the RAS-RAF-MAP kinase (MAPK) pathway via the GTP exchanger SOS, PI3K, PLCγ, and JAK2 results in accumulation of cellular Ca2+ and activation of transcription factors and survival pathways that contribute to differentiation, migration, adhesion, cytokine production and longevity. The SRC kinases LYN and FYN are also involved in these signaling networks. Activation of FcϵRI by a bi- or

multi-valent allergen results in formation of FcϵRI dimers or multimers (not shown in the figure). After translocation to lipid raft micro-domains and the LYN-dependent phosphorylation of specific tyrosines within these motifs, SYK is recruited to the γ chain-ITAMs, resulting in SYK activation and phosphorylation of the transmembrane adaptors LAT and NTAL. Phosphorylated LAT and NTAL coordinate downstream signaling cascades by providing multiple phospho-tyrosine-based docking sites for PLCγ1 and the cytosolic adaptors GRB2, GADS, and SHC. These interactions in turn coordinate the activation of PLCγ1 and PLCγ2, which are required for calcium mobilization and PKC activation. A parallel pathway, initiated by FYN, activates PI3K via the phosphorylation of the cytosolic adaptor GAB2. These events also contribute to degranulation and cytokine production, likely due to PI3K-dependent membrane association of BTK and activation of transcription factors. PI3K may also regulate the activation of sphingosine kinase (SK) and PLD, which produce sphingosine-1-phosphate (S-1-P) and diacylglycerol (DAG). The GTP exchangers SOS and VAV activate the RAS-RAF-MAPK pathway, which contributes to activation of specific transcription factors required for cytokine production. In addition, the MAPKs ERK1/2 mediate activation of PLA2, which liberates arachidonic acid from membrane lipids and thus contribute to generation of eicosanoids. Note that, for clarity, several of the intermediary steps and signaling cascade interactions have been simplified and additional pathways that may down-regulate (or even block) mast cell activation are not included in this figure. Adapted from Metcalfe, Blood. 2008; 112: 946-56.

Figure S2

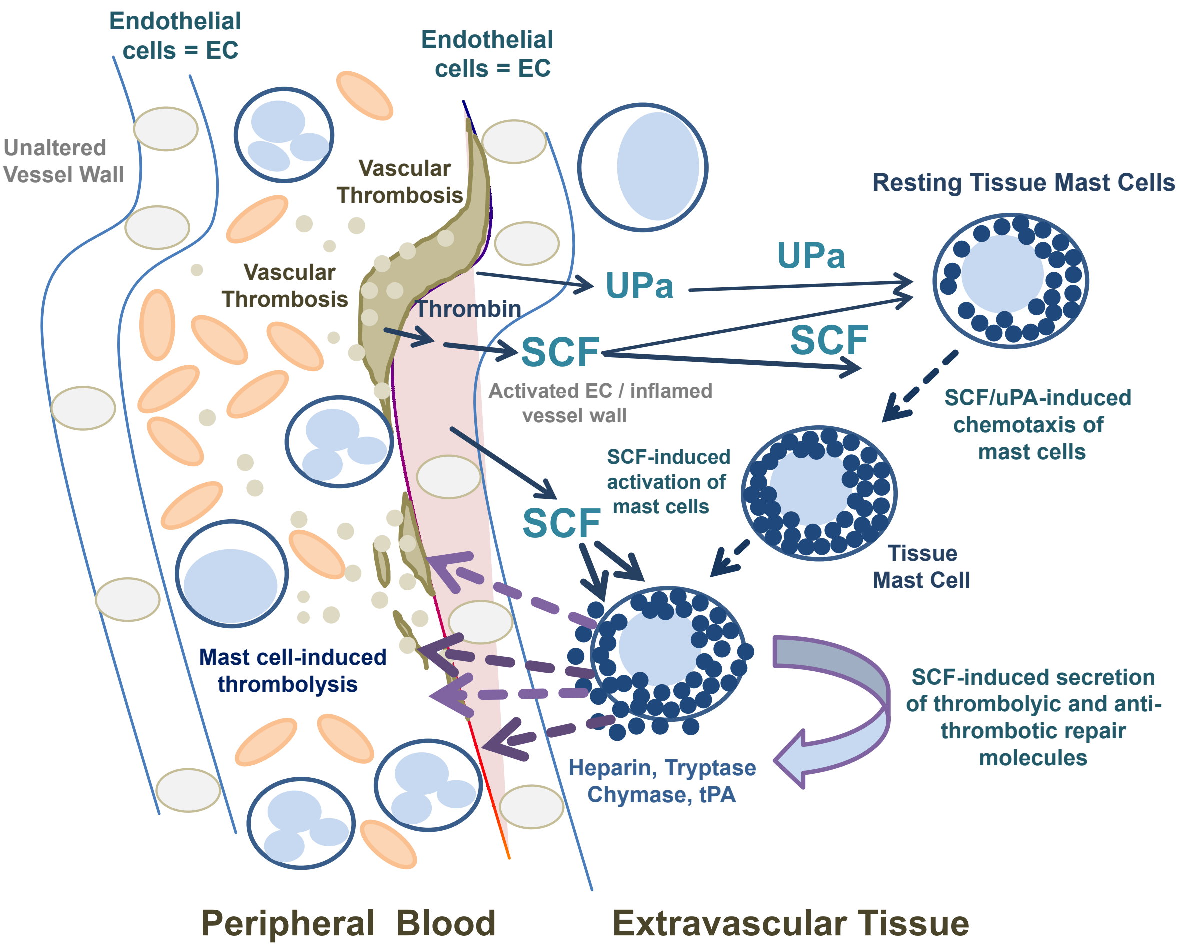

Potential role of mast cells as reparative cells in thromboembolic events

During a thromboembolic event, such as vascular thrombosis, endothelial cells become activated through several mechanisms. These include activation by cytokines and by thrombin. Thrombin-activated endothelial cells (EC) produce and secrete pro-inflammatory molecules and cytokines, including stem cell factor (SCF) and urokinase-type plasminogen activator (uPA). Resting tissue mast cells display receptors for SCF (= KIT/CD117) and uPA (uPAR/CD87). Soluble SCF and uPA are chemotactic for mast cells, thus helping to induce mast cell migration and accumulation at sites of thrombus formation. When the concentration of SCF increases around locally attracted mast cells, these cells can also be activated to release pro-inflammatory molecules, including histamine and molecules with potential repair functions, including heparin, tissue-type plasminogen activator (tPA), tryptase and

chymase. While histamine counteracts vascular occlusion and mediates trans-endothelial migration of cells and repair molecules, heparin and tPA, as well as tryptase and chymase, can counteract further thrombus formation and mediate thrombolysis. In this phase of repair, heparin acts as a co-factor for anti-thrombin III and a co-factor for tPA and fibrinogenolytic tryptase. The accumulation of peri-vascular mast cells in proximity to sites of thrombus formation has been documented in virtually all models of thromboembolism examined, including pulmonary embolism, deep vein thrombosis, and auricular heart thrombosis.

Figure S3

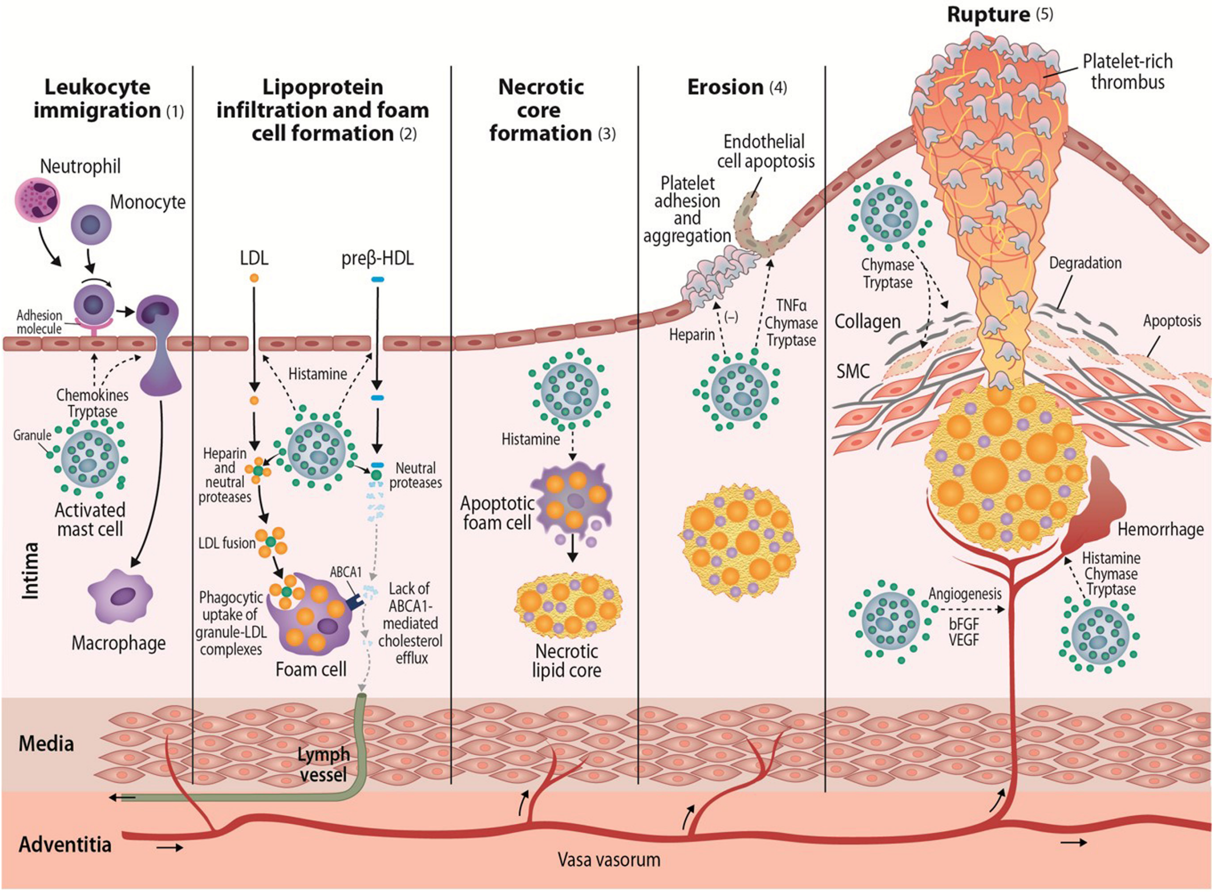

Potential mast cell-mediated effects on initiation and progression of atherosclerotic plaque

Sections 1 through 5, from left to right: 1. Intimal mast cells, when activated, contribute to lesion initiation by inducing leukocyte recruitment via release of chemokines and tryptase, and by enhancing adhesion molecule expression on endothelial cells. 2. Activated mast cells release vasoactive substances, such as histamine, which increase vascular permeability for low-density lipoprotein (LDL) particles and high-density lipoprotein (HDL) particles, among them the discoidal HDL (preβ-HDL). Left: in the sub-endothelial space, LDL particles bind to the heparin component of exocytosed granules. Granule chymase then degrades the apolipoprotein B-100 component of the heparin-bound LDL particles, and thereby triggers particle fusion. Finally, granules covered by fused LDL-particles are phagocytosed by macrophages, which gradually become filled with cytoplasmic lipid droplets

containing LDL-derived cholesterol, transforming them into foam cells. Right: Granule neutral proteases degrade preβ-HDL particles, which thereby lose their ability to accept cholesterol from foam cells via ABCA1 transporter-mediated high-affinity efflux. 3. Histamine may induce apoptosis of macrophage foam cells. Generation of apoptotic bodies, and displacement of the lipid droplets from the cytoplasm into the extracellular space, contribute to the formation of a necrotic lipid core. 4. Release of chymase, tryptase and/or TNFα by activated sub-endothelial mast cells can induce endothelial cell apoptosis. Loss of endothelial cells (erosion) leads to exposure of thrombogenic subendothelial structures, which activate platelets to adhere and cover the exposed area. Mast cells also release macromolecular soluble heparin, which can prevent platelet aggregation and the formation of a larger thrombus. 5. Activated mast cells in the fibrous cap of an atherosclerotic plaque release chymase and tryptase, which activate matrix metalloproteinases. These matrix metalloproteinases then may degrade matrix molecules, such as collagen. Chymase can degrade various components of the pericellular matrix of smooth muscle cells (SMC), notably fibronectin, and thereby may induce their apoptotic death. Activated mast cells located deeper in the plaque, can secrete proangiogenic growth factors, such as basic fibroblast growth factor (bFGF) and vascular endothelial growth factor (VEGF), which induce growth of *vasa vasorum* from the medial layer to the deep hypoxic areas of the plaque. Mast cell-derived histamine, chymase, and tryptase can induce leakage and rupture of the fragile neovascular sprouts, with ensuing intraplaque hemorrhage. Together, collagen degradation, SMC death, and microhemorrhages render the plaque unstable and susceptible to rupture and formation of an arterial platelet-rich thrombus. Adapted from Kovanen and Bot, Eur J Pharmacol. 2017; 816: 37-46.
